# Early porosity generation in organic-sulfur-rich mudstones

**DOI:** 10.1038/s41598-023-35259-5

**Published:** 2023-06-19

**Authors:** Levi J. Knapp, Omid H. Ardakani, Julito Reyes, Kazuaki Ishikawa

**Affiliations:** 1 Japan Organization for Metals and Energy Security (JOGMEC), Chiba, Japan; 2 Natural Resources Canada, Geological Survey of Canada, Calgary, Canada; 3 Department of Geoscience, University of Calgary, Calgary, Canada; 4 Present Address: Alberta Energy Regulator, Edmonton, Canada

**Keywords:** Geochemistry, Geology, Sedimentology

## Abstract

High total organic sulfur (TOS) content (i.e., Type IIS kerogen) is well known to significantly influence kerogen transformation but the effect of TOS content on the evolution of organic porosity has only rarely and indirectly been investigated. This study demonstrates that organic porosity is generated at lower thermal maturity in mudstones containing Type IIS kerogen relative to those with Type II kerogen. To our knowledge this phenomenon has not been previously demonstrated. The implications are relevant for the characterization of organic-rich mudstones as cap rocks, hydrocarbon reservoirs, and disposal reservoirs for CO_2_ or nuclear waste because pore systems control storage volumes and matrix fluid flow. Five thermally immature core samples were selected from three organic-rich mudstone units with low to high TOS content: the late Devonian Duvernay Formation (Canada), middle late Miocene Onnagawa Formation (Japan), and early Jurassic Gordondale member of the Fernie Formation (Canada). Hydrous pyrolysis was used to artificially mature splits of the immature samples to four maturity stages, upon which petrophysical and organic geochemical properties were measured and compared to baseline immature samples. Most porosity growth in Type IIS samples occurred below 0.70% VRo_eqv_, but in Type II samples was broader and robust until 1.1% VRo_eqv_.

## Introduction

Organic matter (OM)-hosted porosity (“organic porosity”) in self-sourced, unconventional shale oil and gas reservoirs typically dominates over inorganic porosity, and as such can critically influence hydrocarbon storage volume, adsorption capacity, permeability, and wettability^1,2^. Organic porosity becomes dominant as burial diagenesis eliminates much of the primary inorganic porosity through compaction, grain re-orientation, ductile deformation^3–5^, and cementation^6–10^, while secondary organic porosity is created through transformation of convertible kerogen and bitumen into liquid and gaseous hydrocarbons^11–18^. Additionally, inorganic pores can be extensively occluded when viscous bitumen and oil migrate into those pores and then undergo secondary thermal cracking into lighter fluid hydrocarbons and residual solid bitumen and pyrobitumen^14,16,19–24^. However, significant variations in organic pore volume, pore size distribution, and morphology have been documented at every observable scale.

The first order control on organic porosity is thermal maturity. Primary kerogen porosity tends to become compacted and/or occluded, but the transformation of kerogen and bitumen into liquid and gaseous hydrocarbons generates extensive secondary porosity in the residual solid OM^11–16,25,26^. The secondary control on organic porosity is effective stress. In the absence of a sufficiently rigid mineral matrix, overburden or tectonic stress will compress OM, drive out liquid hydrocarbons, and reduce OM-hosted porosity^7,25–29^. Lastly, organic porosity development is also influenced by OM type and composition, most easily observed as the absence of secondary porosity in some zooclasts such as chitinozoan and graptolite^30,31^ and terrestrial OM (i.e., vitrinite and inertinite)^32^. There is a wealth of literature demonstrating that high total organic sulfur (TOS) content (i.e., Type IIS kerogen) significantly influences kerogen transformation kinetics^33–39^, however the effect of TOS content on organic porosity evolution has only rarely and indirectly been investigated^40^.

This study investigated the influence of TOS content on the evolution of organic porosity and pore-occluding solid bitumen by using hydrous pyrolysis (HP) to artificially mature a set of immature organic-rich mudstones with a range of TOS content, and subsequently ascertain the changes in their respective geochemical and petrophysical properties. The motivation for this research was to further delineate OM compositional controls on pore system evolution and provide insight for the evaluation of shales with Type IIS kerogen as cap rocks, unconventional hydrocarbon reservoirs, and storage reservoirs for CO_2_ or nuclear waste. Five thermally immature core samples were selected from three organic-rich mudstone units, which in order of increasing TOS content were the late Devonian Duvernay Formation (Canada), middle-late Miocene Onnagawa Formation (Japan), and early Jurassic Gordondale (formerly Nordegg) member of the Fernie Formation (Canada).

## Results

### Characterizing thermally immature samples

#### X-ray diffraction

Mineralogy of immature samples strongly varies between sample families (Table 1). DVRN1 is dominated by quartz and aluminosilicate minerals such as illite and potassium feldspar. DVRN2 is highly calcareous with subsidiary quartz. ONNA is dominated by quartz. GORD1 is calcareous-siliceous. GORD2 is a mixture of quartz, potassium feldspar, and dolomite.

**Table 1:** Results of X-ray diffraction (XRD) mineralogy for immature samples.

Formation	Sample family	Quartz	Plagioclase	K-feldspar	Calcite	Dolomite	Kaolinite	Illite/smectite/mica	Pyrite	Total
		wt%	wt%	wt%	wt%	wt%	wt%	wt%	wt%	wt%
Duvernay	DVRN1	32.5	–	22.3	15.7	–	3.6	22.6	3.3	100.0
Duvernay	DVRN2	18.6	–	–	57.2	9.3	3.7	10.4	0.9	100.1
Onnagawa	ONNA	88.1	–	–	–	–	–	10.3	1.6	100.0
Gordondale	GORD1	28.7	–	12.0	29.5	16.6	–	11.1	2.0	99.9
Gordondale	GORD2	34.9	4.1	27.3	–	20.9	–	11.1	1.7	100.0

#### Total sulfur speciation and programmed pyrolysis

**Figure 1:**
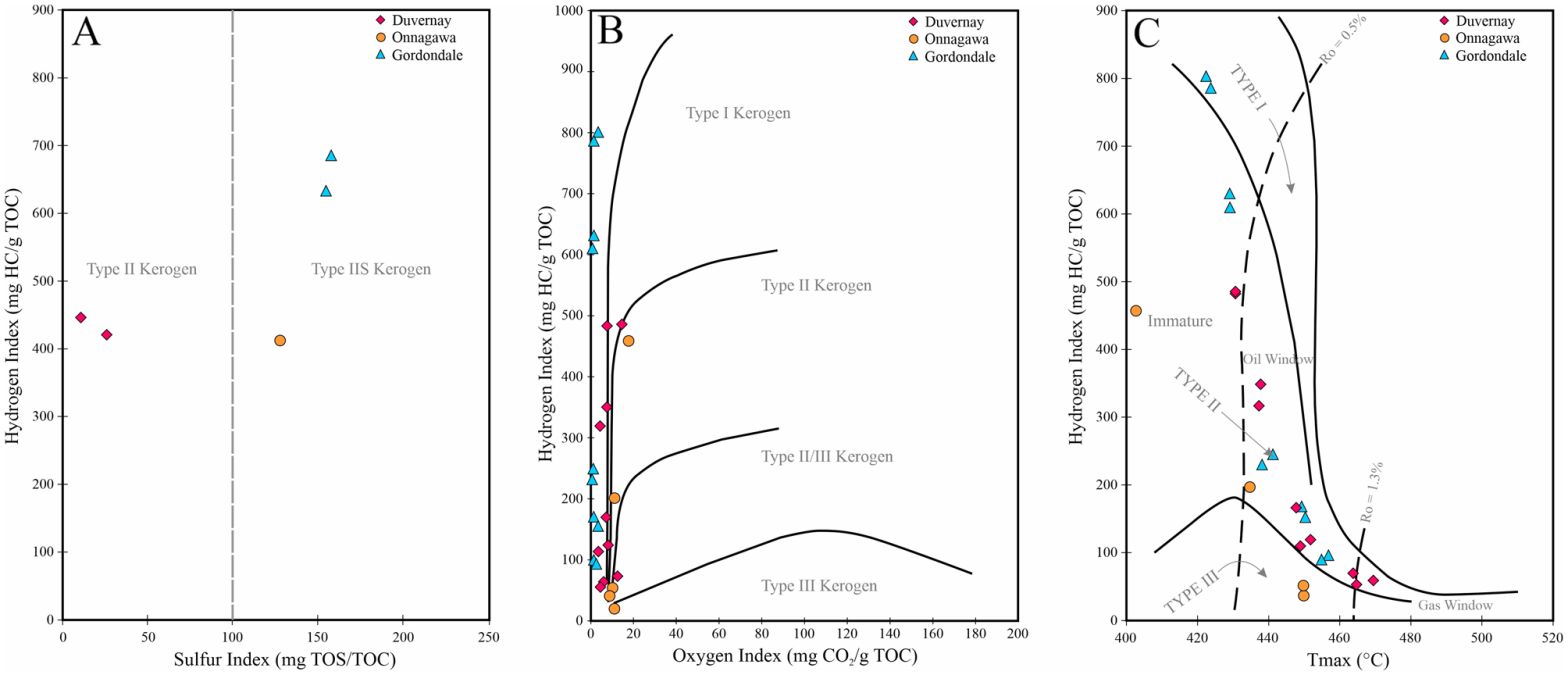
(**A**) Hydrogen index (HI) versus sulfur index (SI) of the studied samples based on sulfur speciation using Rock–Eval 7S. Gordondale and Onnagawa samples contain organic matter characterized as Type IIS kerogen while Duvernay organic matter is Type II kerogen. (**B**) Hydrogen index (HI) versus oxygen (OI). (**C**) HI versus Tmax plots of the studied samples. All original samples contain immature marine kerogen.

Total sulfur (TS), total organic sulfur (TOS), sulfur associated with pyrite (Fe-S), and sulfur associated with kerogen (i.e., S2-OS) were measured for all samples (Table 2) using programmed pyrolysis (Rock-Eval 7S). The calculated sulfur index (SI) varies from 10.5 to 158.1 (mg TOS/TOC) which classifies Gordondale (GORD) and Onnagawa (ONNA) samples as sulfur-rich kerogen (Type IIS) and Duvernay (DVRN) samples as Type II kerogen (Fig. 1A). The ONNA immature sample has the lowest TOC content (3.01 wt%) among all samples. DVRN and GORD samples contain 4.34 and 7.46 wt%, 20.61 and 24.17 wt% TOC, respectively (Table 3). The hydrogen (HI) and oxygen (OI) indices of the initial immature samples vary from 459 to 803 mg HC/g TOC and 1 to 18 mg CO_2_/g TOC, respectively indicating marine-sourced OM (Fig. 1B). The Tmax of the initial samples varies from 403 to 431 °C indicating all initial samples contain thermally immature marine kerogen (Fig. 1C).

**Table 2:** Sulfur content of organic and inorganic phases of studied samples measured by Rock-Eval7S programmed pyrolysis.

Formation	Sample family	Depth	TS	TOS	S2-OS	Residual-OS	Fe-S	Carbonates	Pyrite	Sulfur Index (SI)
		m	wt%	wt%	wt%	wt%	wt%	wt%	wt%	PyTOSx1000/TOC
Duvernay	DVRN1	2413.15–2413.30	3.82	0.78	0.19	0.58	1.91	10.00	3.80	25.61
Duvernay	DVRN2	2414.00–2414.40	1.17	0.20	0.10	0.15	0.24	57.42	0.48	10.50
Onnagawa	ONNA	16.57–16.73	2.58	0.52	0.39	0.10	1.72	0.58	3.43	127.73
Gordondale	GORD1	924.70–924.85	4.95	2.66	2.58	0.02	0.91	32.17	1.81	158.08
Gordondale	GORD2	926.00–926.15	5.42	3.42	2.96	0.41	1.53	19.00	3.05	154.99

**Table 3:** Basic programmed pyrolysis results and estimated S2-loss porosity (S2-loss Ø) of immature and artificially-matured samples.

Sample family	Hydrous pyrolysis temperature and duration	S1	S2	PI	S3	Tmax	PC	TOC	RC	HI	OI	PC/TOC	VR_eqv_	S2-loss Ø
		mg HC/g	mg HC/g	S1/(S1 + S2)	mg CO_2_/g	°C	wt%	wt%	wt%	mg HC/g TOC	mg CO2/g TOC			vol%
DVRN1	Immature	3.34	36.04	0.08	0.61	431	3.37	7.46	4.09	483	8	0.45	0.65	
	310 °C × 3 days	1.41	19.15	0.07	0.31	438	1.78	6.01	4.24	319	5	0.30	0.75	4.2
	340 °C × 3 days	3.13	6.51	0.32	0.28	449	0.86	5.79	4.94	112	4	0.15	0.92	3.1
	350 °C × 3 days	0.67	3.02	0.18	0.31	465	0.35	5.45	5.11	55	5	0.06	1.17	0.9
	350 °C × 9 days	2.92	3.21	0.48	0.40	470	0.56	5.17	4.62	62	7	0.11	1.24	0.0
DVRN2	Immature	2.28	21.11	0.10	0.66	431	2.02	4.34	2.32	486	15	0.47	0.65	
	310 °C × 3 days	0.61	12.23	0.05	0.30	438	1.12	3.49	2.37	351	8	0.32	0.75	2.2
	340 °C × 3 days	1.48	5.50	0.21	0.26	448	0.62	3.26	2.63	169	8	0.19	0.91	1.7
	350 °C × 3 days	1.37	3.74	0.27	0.27	452	0.46	3.05	2.59	122	9	0.15	0.97	0.4
	350 °C × 9 days	1.01	2.17	0.32	0.40	464	0.30	2.99	2.69	73	13	0.10	1.15	0.4
ONNA	Immature	0.35	13.85	0.02	0.56	403	1.24	3.01	1.78	459	18	0.41	0.22	
	310 °C × 3 days	0.38	4.65	0.08	0.28	435	0.45	2.33	1.88	200	12	0.19	0.71	2.3
	340 °C × 3 days	0.28	1.13	0.20	0.24	450	0.15	2.08	1.94	54	11	0.07	0.94	0.9
	350 °C × 3 days	0.32	0.94	0.26	0.23	450	0.13	2.34	2.20	40	9	0.06	0.94	0.0
	350 °C × 9 days	0.22	0.45	0.33	0.27	561	0.08	2.23	2.15	20	11	0.04	2.63	0.1
GORD1	Immature	5.52	165.62	0.03	0.93	423	14.59	20.61	6.02	803	4	0.71	0.53	
	310 °C × 3 days	24.73	118.13	0.17	0.45	430	12.17	18.70	6.54	632	2	0.65	0.62	11.8
	340 °C × 3 days	65.93	42.02	0.61	0.26	439	9.20	18.08	8.88	232	1	0.51	0.76	19.0
	350 °C × 3 days	9.73	14.43	0.40	0.42	451	2.08	9.30	7.22	155	4	0.22	0.95	6.9
	350 °C × 9 days	8.41	8.46	0.50	0.33	455	1.47	9.03	7.57	93	3	0.16	1.01	1.5
GORD2	Immature	6.61	190.35	0.03	0.68	424	16.78	24.17	7.39	787	2	0.69	0.54	
	310 °C × 3 days	21.47	117.39	0.15	0.29	430	11.83	19.21	7.38	611	1	0.62	0.62	18.2
	340 °C × 3 days	23.26	32.04	0.42	0.27	442	4.72	12.94	8.21	248	2	0.36	0.81	21.3
	350 °C × 3 days	18.79	20.64	0.48	0.32	450	3.38	12.11	8.74	170	2	0.28	0.93	2.8
	350 °C × 9 days	9.82	10.61	0.48	0.28	457	1.76	10.63	8.86	99	2	0.17	1.04	2.5

S2-loss Ø is vol% change in S2 in each sample relative to the preceding sample in the maturity series. S2_wt%_ = S2 × 0.083^41^. S2_vol%_ = S2_wt%_ × 3 (assumes constant S2 OM density = 1/3 of matrix density).

#### Organic petrology

##### Onnagawa Formation

**Figure 2:**
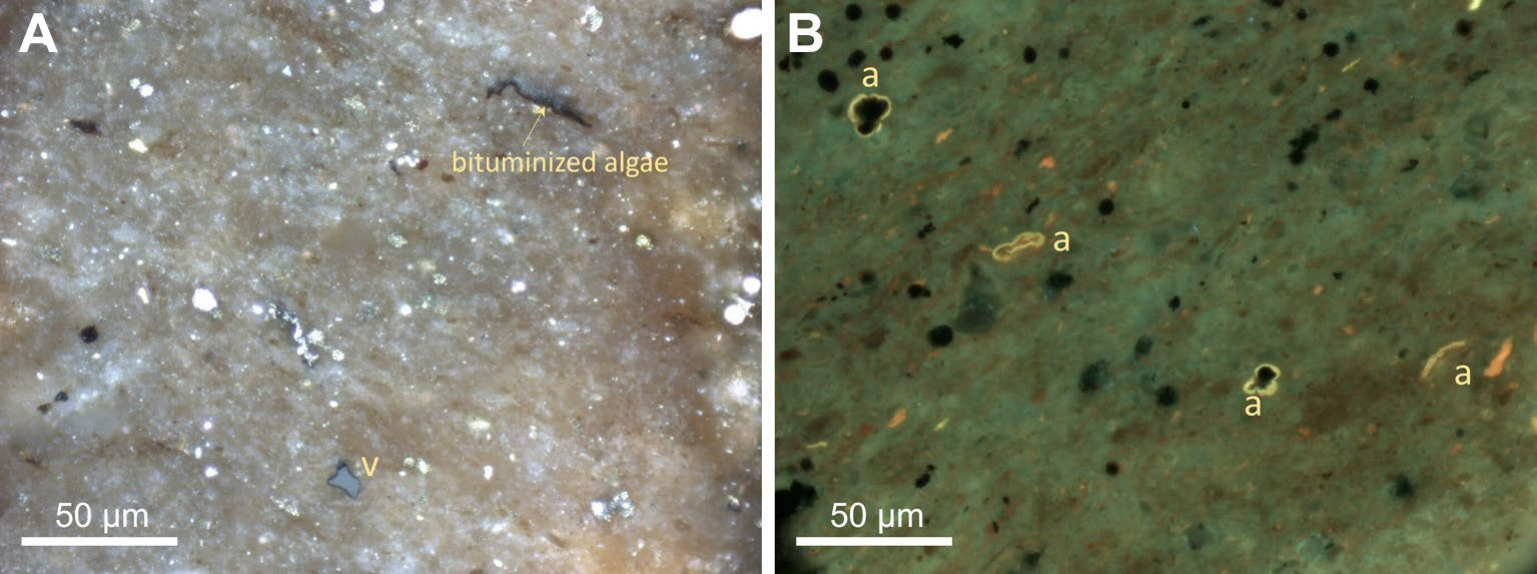
Photomicrographs of Onnagawa immature samples with incident white (left) and ultraviolet (UV) light (right) under oil immersion with × 50 objective. (**A**) a finely crystalline rock matrix with sporadic fine pyrite crystals, partially spent algae and vitrinite (v) particles, and (**B**) sporadic green to yellow fluorescing alginite (a) macerals.

The dominant organic macerals in the Onnagawa sample (Fig. 2) are alginite with minor vitrinite. Some alginite were transformed *in-situ* to bituminite and solid bitumen while the majority of alginite show green to yellow fluorescence color, which indicates low thermal maturity. The average random solid bitumen reflectance (BRo) is 0.22 ± 0.07% (n = 21) which is equal to 0.54% equivalent vitrinite reflectance (VRo_eqv_), calculated using Jacob’s equation^42^. Both the observed fluorescence color and measured reflectance indicates the sample is immature.

##### Duvernay Formation

**Figure 3:**
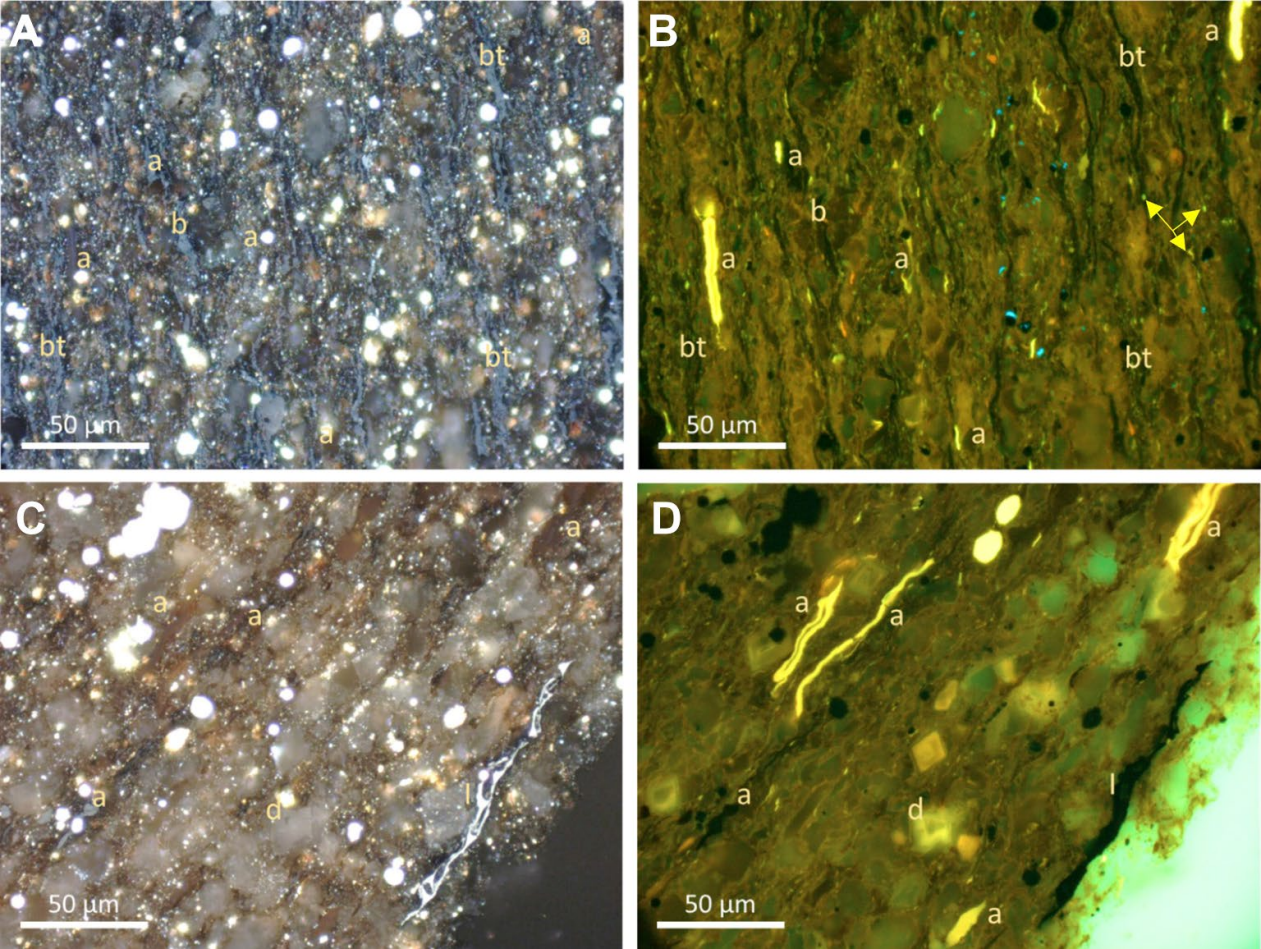
Photomicrographs of Duvernay immature samples with incident white (left) and ultraviolet (UV) light (right) under oil immersion with × 50 objective. (**A**, **B**) Pair view shows a fine-grained clay-rich matrix with long thin lenses of weak reddish-orange fluorescing bituminite (bt) and traces of solid bitumen (b), thin long and short lenses of greenish yellow to reddish orange fluorescing alginite (a) and liptodetrinite (arrow). (**C**, **D**) Pair view shows coarser matrix with less clay, minor amount of thin long and short lenses of greenish yellow to reddish orange fluorescing alginite (a), and traces of inertinite. Dolomite crystals (d) were also observed.

The dominant OM in the immature Duvernay samples (Fig. 3) are bituminite (as *in-situ* bituminized algae), alginite, minor inertinite and chitinozoan fragments, and pore-filling solid bitumen^28,43^. Alginite exhibits green to yellow fluorescence color, which indicates low thermal maturity (Fig. 3). The BRo of DVRN1 and DVRN2 is 0.33 ± 0.07% (n = 150) and 0.41 ± 0.08 (n = 105), respectively. The VRo_eqv_ of DVRN1 and DVRN2 is 0.60 and 0.65% (Table 3).

##### Gordondale member

**Figure 4:**
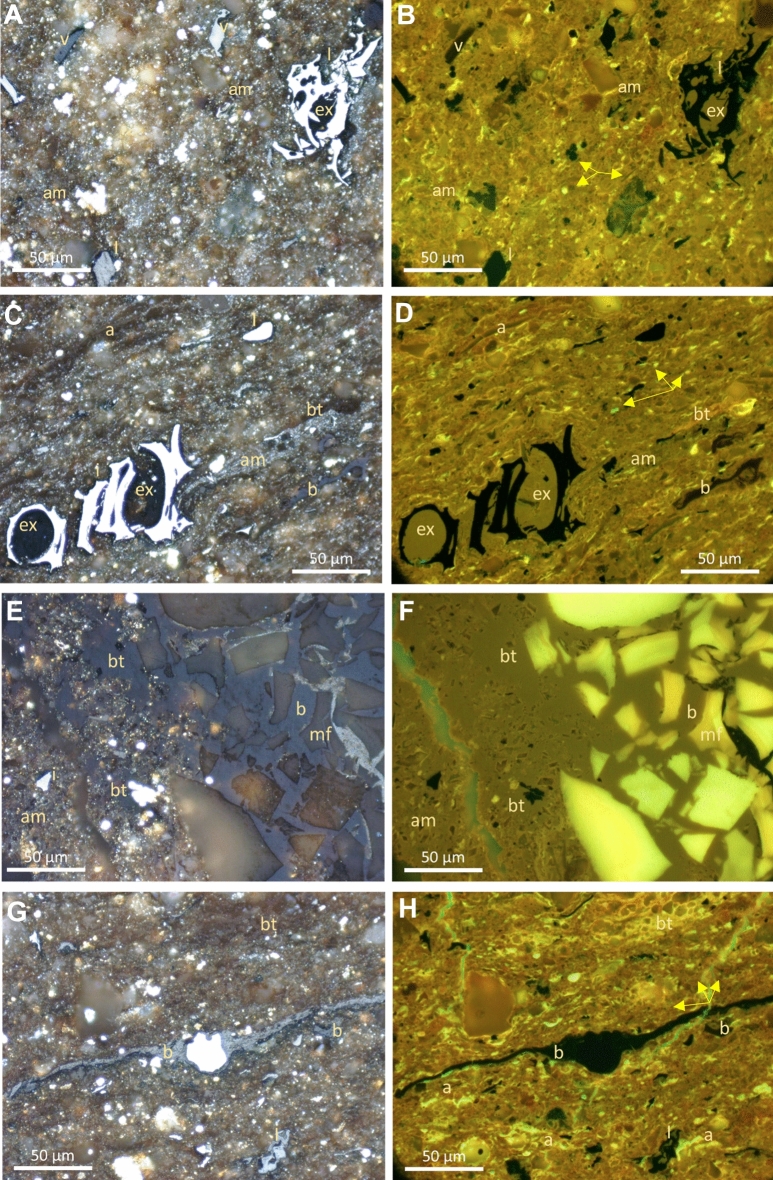
Photomicrographs of Gordondale immature samples observed under incident white (left) and ultraviolet (UV) light (right) under oil immersion with × 50 objective. (**A**, **B**) Pair view of abundant brown amorphous kerogen (am) and greenish-yellow to yellow fluorescing elongated thin lenses of alginite (a) and liptodetrinite (arrows), and traces of vitrinite (v), inertinite (I) and dark orange fluorescing exsudatinite (ex). (**C**, **D**) Pair view shows a laminated matrix with large orange to reddish-brown fluorescing alginite (a) and solid bitumen (b), thin lenses of greenish-yellow to yellow fluorescing alginite (a) and liptodetrinite (arrow), and weak fluorescing exudatinite (ex). (**E**, **F**) Pair view shows reddish-orange fluorescing matrix consisting of amorphous kerogen (am) bituminite (bt) and solid bitumen (b) brecciated between bright fluorescing calcareous microfossil (mf) fragments. (**G**, **H**) Pair view shows matrix with large long lenses of brown-fluorescing and small reddish-brown-fluorescing solid bitumen (b), yellow fluorescing bituminite (bt), long thin lenses of greenish-yellow to yellow fluorescing alginite (a) and liptodetrinite (arrow), traces of inertinite (I).

In comparison to ONNA and DVRN samples, GORD1 and GORD2 are very organic-rich with higher maceral type variation. The dominant macerals are alginite, bituminite, vitrinite, inertinite, solid bitumen, and exsudatinite (Fig. 4). Exsudatinite and solid bitumen mainly fill the pore space within inertinite, intergranular pores, and microfossils (Fig. 4A–F). Abundant exsudatinite and matrix bitumen (i.e., pre-oil bitumen) with dark yellow brown fluorescence were observed in both samples (Fig. 4). Alginite also show bright yellow fluorescence which indicates low thermal maturity (Fig. 4). In some cases, exsudatinite turned into solid bitumen with a polishable surface that could be distinguished under white incident light (Fig. 4E,F). There is also abundant solid bitumen, formed *in-situ* by the thermal degradation and transformation of alginite macerals (Fig. 4G,H). The BRo of GORD1 and GORD2 samples is 0.14 ± 0.04% (n = 145) and 0.27 ± 0.08% (n = 81), respectively, which has VRo_eqv_ of 0.49 and 0.57% (Table 3).

### Geochemical changes during maturation

#### Programmed pyrolysis and organic petrology

Hydrous pyrolysis stages were conducted at isothermal temperature of 310, 340, 350 (for 3 days) and 350 °C (for 9 days) and are referred to in the text as 310 × 3, 340 × 3, 350 × 3, and 350 × 9. The variations of programmed pyrolysis parameters over the HP series of all samples are shown in Fig. S1 online. Except for S1 (free hydrocarbon) that is subject to evaporative loss (e.g.,^45^) during sample handling and preparation, the other parameters show a clear trend with increasing thermal maturity during artificial maturation (Table 2). The S2 (kerogen content) and HI show a consistent and sharp decline with thermal maturity advancement, while Tmax values increased consistently with increasing temperature. The ONNA sample at 350 × 9 shows anomalously high Tmax (561 °C) which is due to a low S2 yield (0.45 mg HC/g) (e.g.,^46,47^). This high value caused a significant increase in the Tmax-derived Ro value (2.63%) (Table 2). The TOC content of all samples except ONNA shows a consistent decreasing trend with thermal maturity advancement, which is consistent with thermal degradation and conversion of convertible OM into various hydrocarbon fractions.

#### Onnagawa Formation

The physicochemical transformation of kerogen in ONNA samples started from 310 × 3. At this stage, some of the easily convertible OM such as bituminite and alginite started to thermally transform *in-situ* to various hydrocarbon fractions (oil, viscous and solid bitumen and gas). Alginite (*Tasmanites*) with greenish fluorescence color started to change from green to dark yellow and produce small quantites of greenish-yellow fluorescing oil oozing onto the surface from the thermally degraded alginite (observed under UV light). The VRo_eqv_ increased slightly at this stage, from 0.54 to 0.68%, which is in accordance with an increase in Tmax from 403 to 435 °C (Table 4). At 340 × 3, most of the alginite transformed *in-situ* to oil, viscous and solid bitumen and its UV fluorescence color turned to dark red or in some cases totally diminished. VRo_eqv_ further increased to 1.10% with increase in Tmax from 435 to 447 °C (Table 4, Fig. S1). At 350 × 3 almost all alginite were thermally converted to solid bitumen. VRo_eqv_ increased slightly to 1.19% while the Tmax did not change. At the final stage of HP analysis (350 × 9), VRo_eqv_ further increased to 1.45%, with unreliable Tmax-derived VRo_eqv_ due to low S2 yield (Table 3).

**Table 4:** Measured vitrinite reflectance, bitumen reflectance and %VR equivalent, and %VRo from Tmax.

Sample family	HP temperature and duration	Organic matter type							^#^Measured Tmax	VR_eqv_ from Tmax
		Vitrinite			Bitumen			VR_eqv_		
		%Ro	SD	N	%Ro	SD	N	%Ro	°C	%Ro^1^
DVRN1	Immature	na	na	na	0.33	0.07	150	**0.60**	431	0.60
	310 °C × 3 days	0.77	0.07	23	0.54	0.08	18	**0.74**	438	0.72
	340 °C × 3 days	1.08	0.10	11	1.06	0.20	40	**1.06**	450	0.94
	350 °C × 3 days	na	na	na	1.41	0.17	50	**1.27**	463	1.17
	350 °C × 9 days	na	na	na	1.54	0.17	51	**1.35**	471	1.32
DVRN2	Immature	na	na	na	0.41	0.08	105	**0.65**	431	0.60
	310 °C × 3 days	0.72	0.08	45	0.46	0.10	24	**0.69**	438	0.72
	340 °C × 3 days	1.08	0.06	12	1.13	0.15	44	**1.10**	450	0.94
	350 °C × 3 days	1.18	0.07	5	1.40	0.14	49	**1.27**	454	1.01
	350 °C × 9 days	na	na	na	1.65	0.18	76	**1.42**	504	1.91
ONNA	Immature	na	na	na	0.22	0.06	21	**0.54**	403	0.09
	310 °C × 3 days	0.71	0.05	14	0.45	0.09	16	**0.68**	435	0.67
	340 °C × 3 days	1.20	0.02	2	1.14	0.11	29	**1.10**	447	0.88
	350 °C × 3 days	na	na	na	1.28	0.18	37	**1.19**	446	0.87
	350 °C × 9 days	na	na	na	1.64	0.15	48	**1.41**	562	2.96
GORD1	Immature	na	na	na	0.14	0.04	145	**0.49**	423	0.45
	310 °C × 3 days	na	na	na	0.72	0.09	33	**0.70**	430	0.58
	340 °C × 3 days	0.90	0.08	13	0.82	0.05	13	**0.90**	446	0.87
	340 °C × 3 days	*na*	*na*	*na*	*0.52*	*0.12*	*40*	*0.72*	*446*	*0.87*
	350 °C × 3 days	0.97	0.07	12	1.11	0.13	36	**1.08**	454	1.01
	350 °C × 9 days	1.25	0.02	2	1.56	0.18	56	**1.37**	464	1.19
	350 °C × 9 days	*na*	*na*	*na*	*1.73*	*0.44*	36*	*1.47*	*464*	*1.19*
GORD2	Immature	na	na	na	0.27	0.08	81	**0.57**	424	0.47
	310 °C × 3 days	0.72	0.06	18	0.42	0.10	8	**0.66**	433	0.63
	340 °C × 3 days	0.90	0.12	11	0.98	0.12	31	**1.01**	443	0.81
	350 °C × 3 days	1.13	0.06	10	1.10	0.11	38	**1.08**	453	0.99
	350 °C × 9 days	1.25	0.08	3	1.53	0.14	69	**1.35**	470	1.30

Bolded %VRo_eqv_ values were used for plotting against porosity data. Italicized data indicate secondary populations of bitumen. na: no measured vitrinite particles, ^#^average of two to three measurements, *anisotropic fine-grained mosaic pyrobitumen, ^1^VR_eqv_ based on Tmax^44^.

#### Duvernay Formation

The physicochemical transformation of kerogen in DVRN1 and DVRN2 samples started from 310 × 3. At this stage, greenish-yellow fluorescing alginite in the immature samples started to transform *in-situ* into various hydrocarbon fractions resulting from the thermal degradation and conversion of OM. The VRo_eqv_ of DVRN1 and DVRN2 increased from 0.60 and 0.65 to 0.74 and 0.69%, respectively. The Tmax value and Tmax-derived VRo_eqv_ also showed the same increasing trend (Table 4, Fig. S1). At 340 × 3 the fluorescence color of alginite continued to shift from yellow to dark yellow to orange with further thermal cracking. Generated solid bitumen started to fill pores within the matrix and OM. The first evidence of generated oil was observed under UV light. The amount of solid bitumen and produced oil observed in DVRN samples is significantly higher than ONNA samples. The VRo_eqv_ of DVRN1 and DVRN2 increased to 1.06 and 1.10%, respectively. At 350 × 3, almost all alginite and amorphous OM were completely transformed to various hydrocarbon fractions dominated by oil and solid bitumen. The VRo_eqv_ increased to 1.27 and 1.19% for DVRN1 and DVRN2, respectively (Table 3, Fig. S1). At 350 × 9, the fluorescence color of alginite completely diminished and the adsorbed oil exuded onto the sample surface when exposed to UV light. Solid bitumen was the most observed maceral at 350 × 9. VRo_eqv_ increased to 1.35 (DVRN1) and 1.42% (DVRN2).

#### Gordondale member

The physicochemical transformation of kerogen in GORD1 and GORD2 samples started from 310 × 3 (Tmax = 430–433 °C). Alginite and matrix bitumen transformed to lighter hydrocarbons and the fluorescence intensity of residual alginite and produced solid bitumen were masked by excessive amounts of greenish-yellow fluorescing oil oozing onto the surface of the samples under UV light. GORD samples were significantly richer in inertinite compared to other samples. The VRo_eqv_ of GORD1 and GORD2 increased from 0.49 and 0.57 to 0.70 and 0.66%, respectively. At 340 × 3 the fluorescence color of alginite and matrix bitumen shifted from bright yellow to slightly darker yellow while most of the matrix bitumen and exudatinite transformed to solid bitumen. The VRo_eqv_ of both GORD1 and GORD2 increased to 0.90 and 1.01%, respectively (Table 3). A second BRo population was observed with a lower value (GORD1: 0.52 ± 0.12; n = 40) than the main population (0.82 and 0.98% BRo in GORD1 and GORD2), consistent with scanning electron microscopy (SEM) observations that show two phases of solid bitumen (described below). At 350 × 3 the VRo_eqv_ of both GORD1 and GORD2 increased to 1.08% (Table 3). At 350 × 9, more solid bitumen formed with the advancement in OM thermal cracking. The VRo_eqv_ of GORD1 and GORD2 increased to 1.37 and 1.35%, respectively (Table 4).

### Textural changes during maturation

#### SEM observations

##### Onnagawa Formation

**Figure 5:**
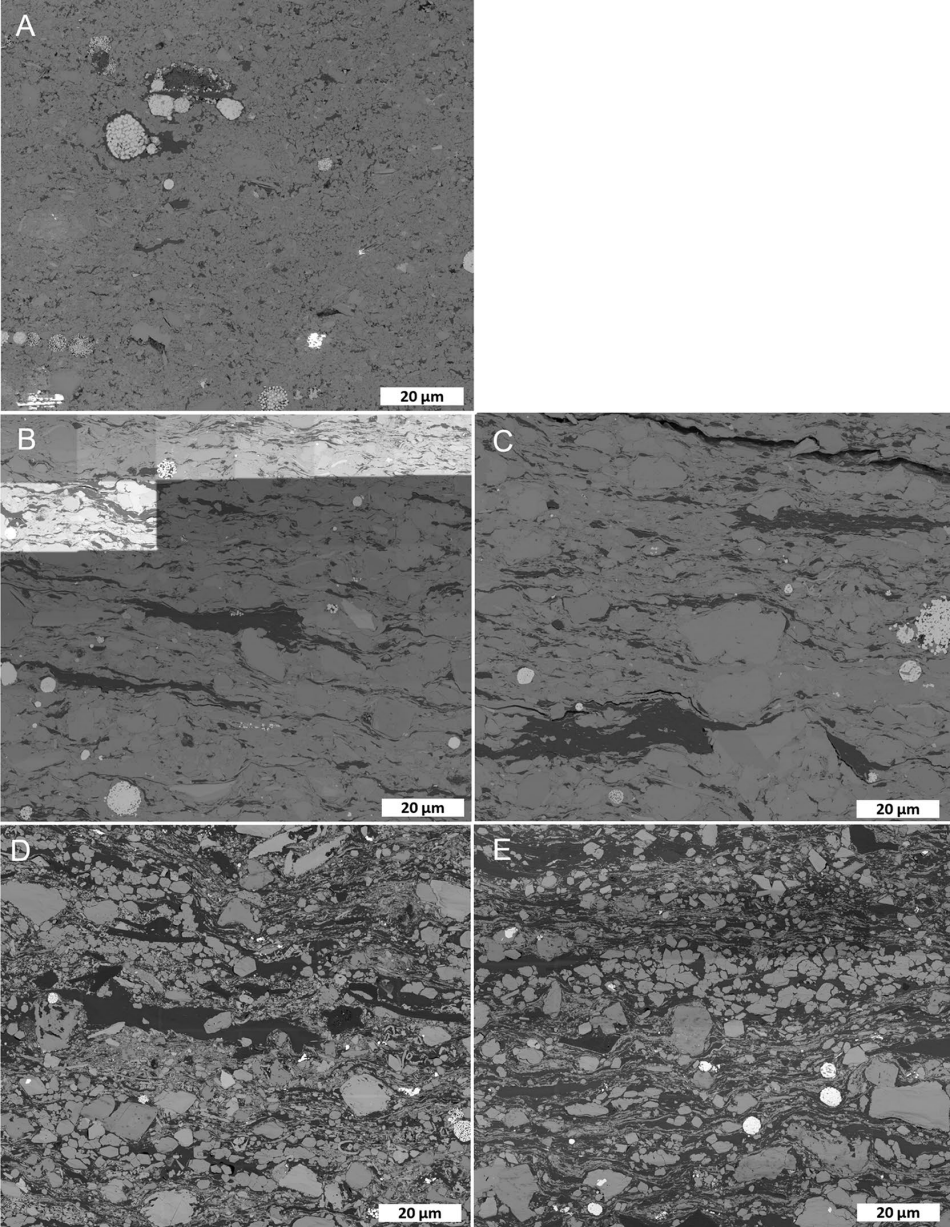
Backscattered electron SEM images of thermally immature samples from all sample families at overview resolution (115 nm/px). Organic matter is dark grey in the images. (**A**) ONNA. (**B**) DVRN1. (**C**) DVRN2. (**D**) GORD1. (**E**) GORD2.

**Figure 6:**
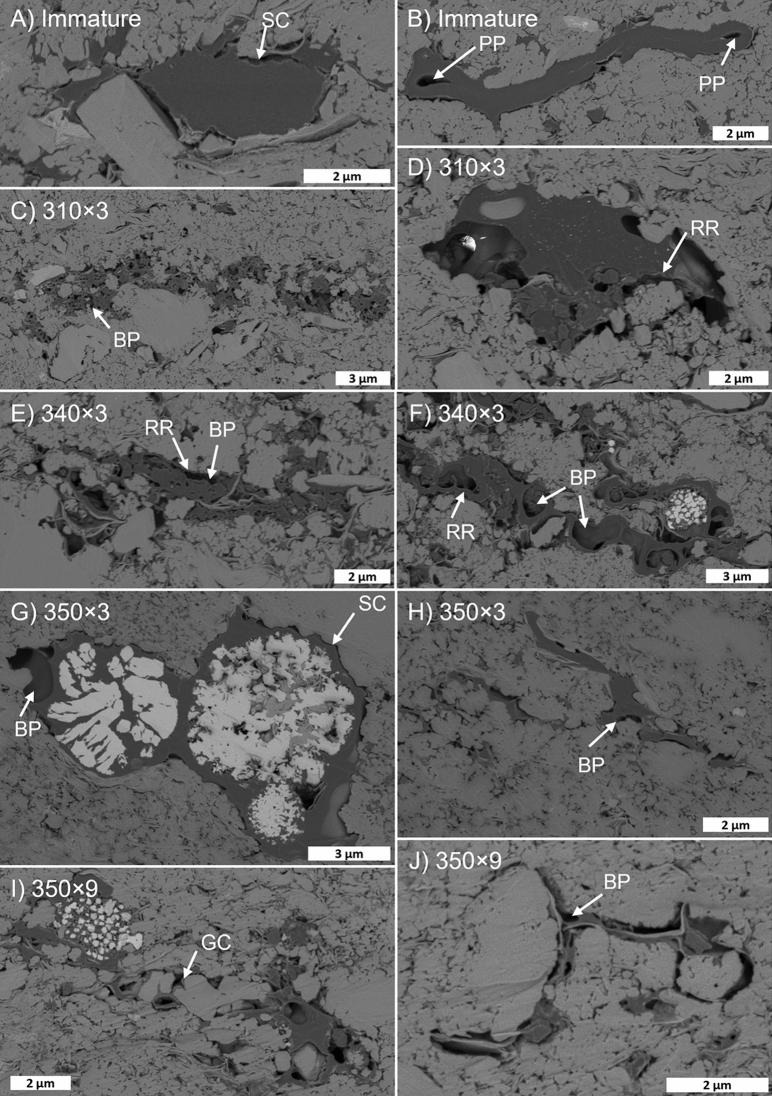
Onnagawa maturity series in high resolution (5 nm/px) backscattered electron SEM images. SC: shrinkage crack; PP: primary pore; BP: bubble pore; RR: raised rim; GC: grain coat.

ONNA samples do not display significant bedding textures at SEM-scale (~ 100’s µm) but typically have isolated OM particles and pyrite framboids dispersed in a porous microcrystalline quartz matrix (Fig. 5A). Pyrite framboids are often hosted within OM, with common signs of plucking. Intercrystalline pores in the microcrystalline quartz matrix are typically 10’s of nm in diameter and are variably filled with OM (Fig. 6). Observable OM porosity in the immature sample (Fig. 6A) is limited to minor primary porosity, as well as shrinkage cracks at mineral–OM interfaces. Bubble-type pores, typically less than 1 µm in diameter, often occurring at mineral-OM interfaces, and sometimes exhibiting raised rims, become common at 310 × 3 and extensive by 340 × 3 (Fig. 6B,C). Bubble pores seem to be less extensive at 350 × 3, however large OM particles are limited (Fig. 6D). Bubble-type pores are common at 350 × 9 (Fig. 6E). From 340 × 3 onwards OM pore diameters are mostly limited by adjacent mineral boundaries and many OM particles exist only as residual grain-coating layers.

##### Duvernay Formation

Organic matter in DVRN samples occurs as large, elongate particles (DVRN1: 10’s-100’s µm long by < 20 µm thick; DVRN2: < 100 µm long by < 10 µm thick) and as intimate mixtures with matrix minerals (Fig. 5B,C). Observable matrix porosity is minor in immature DVRN samples and is restricted to nm-scale pores in tight clusters of clay minerals. Artificially-matured samples contain inorganic porosity within masses of fibrous Mg-rich (as confirmed by SEM–EDS; Fig. S2) diagenetic minerals, altered from dolomite. Intergranular pores between the fibers of these masses are typically 10’s of nm in diameter but likely range from a few nm to a few hundred nm.

**Figure 7:**
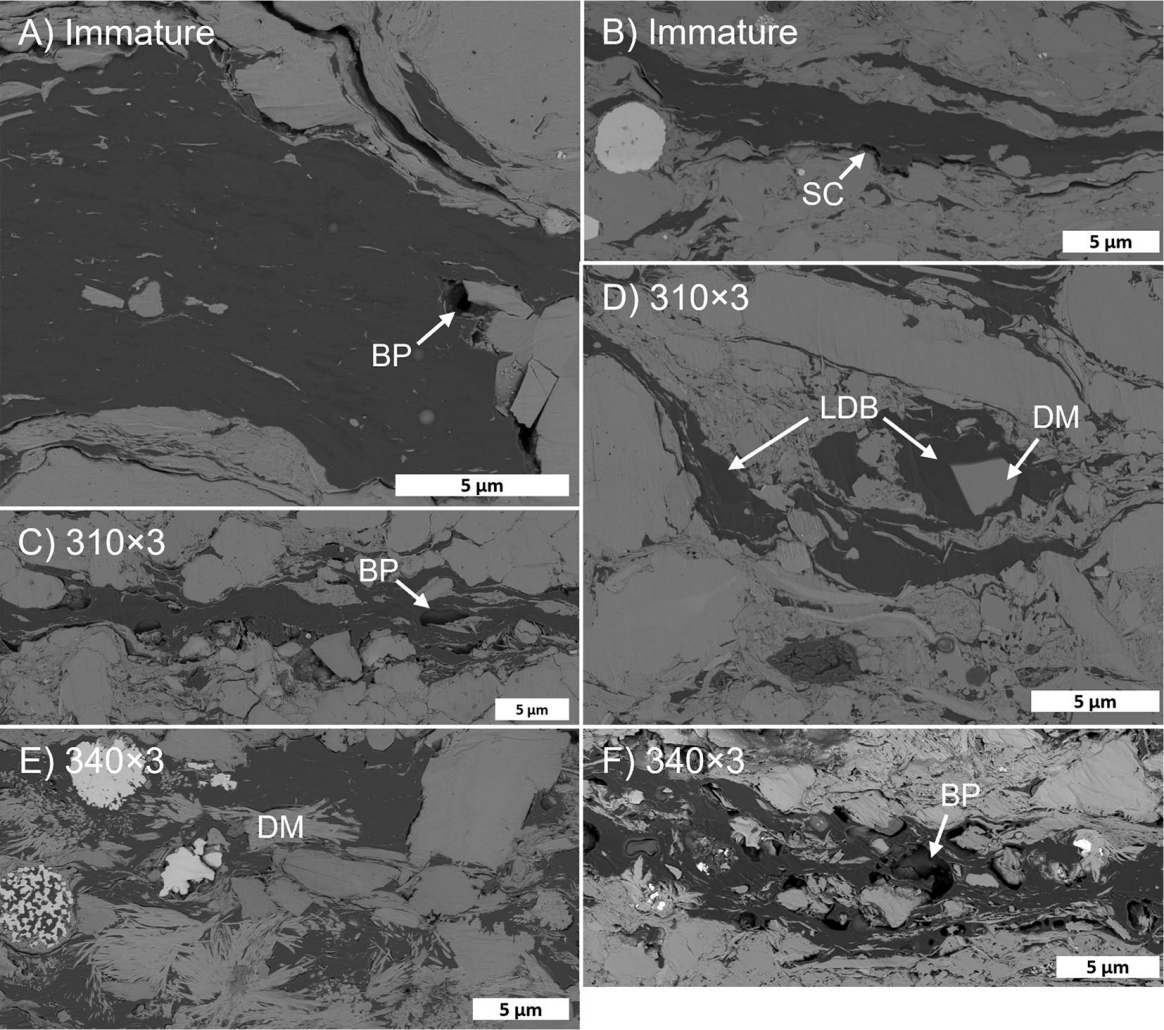

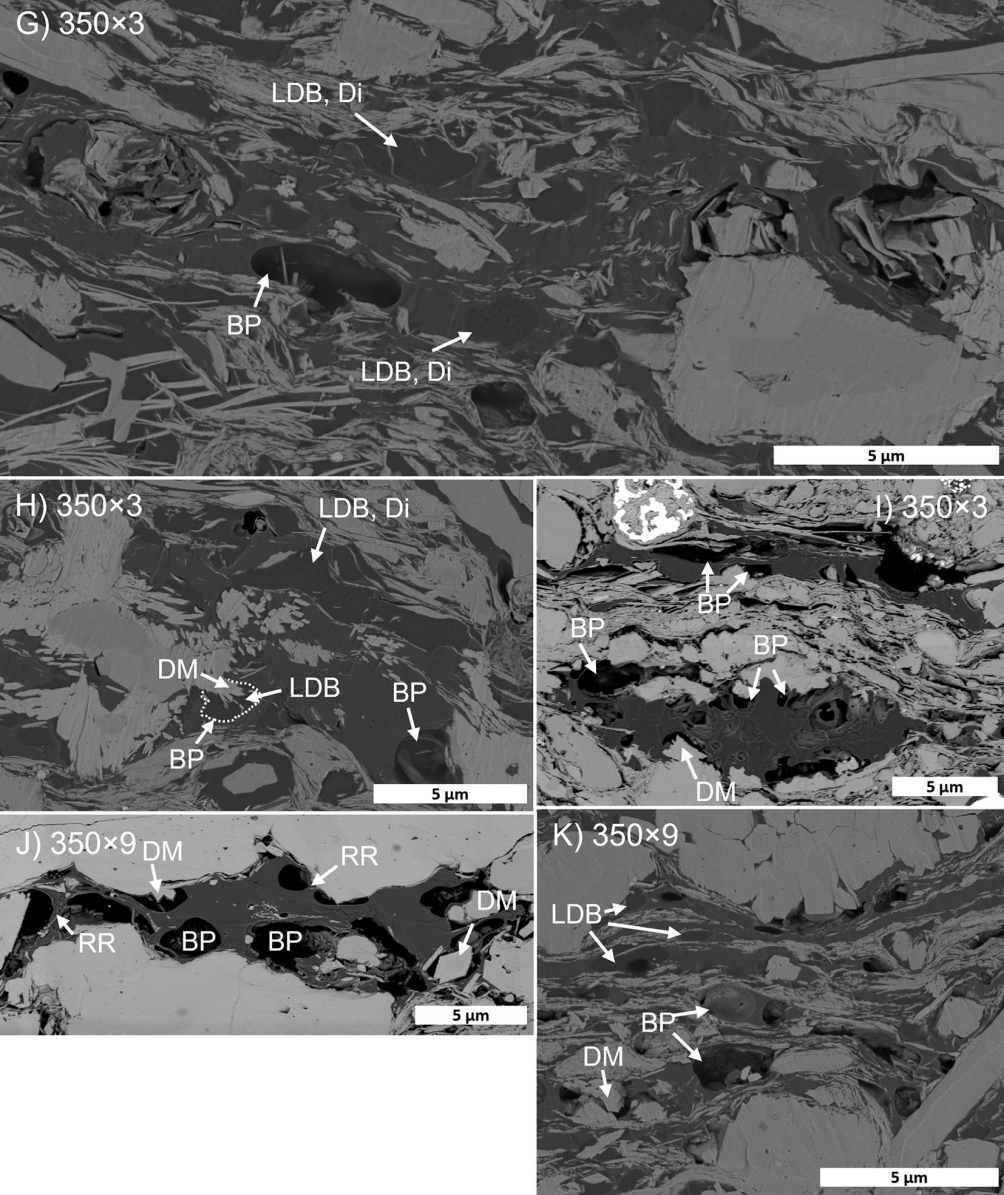
Duvernay maturity series in high resolution (5 nm/px) backscattered electron SEM images. SC: shrinkage crack; BP: bubble pore; RR: raised rim; GC: LDB: low density bitumen; Di: dimples; DM: diagenetic mineral.

Observable OM porosity is nearly absent in the immature DVRN samples, except for shrinkage cracks and very rare bubble pores (Fig. 7A,B). The first widespread appearance of organic porosity is at 310 × 3, and intimate association of OM and authigenic minerals starts at this stage (Fig. 7C). Authigenic minerals often extend into (or are enveloped by) bubble-shaped pores, which may be anchored on mineral surfaces, sometimes have raised rims, and typically have diameters < 3 µm. Bubble pores increase in abundance to 340 × 3 (Fig. 7F), beyond which their occurrence decreases moderately (Fig. 7G–K). Two phases of bitumen, based on greyscale (average atomic mass but interpreted as density; see Methods) contrast, were observed throughout the maturity series. In immature samples irregular to wispy density variations are present within some OM particles (Fig. 7A). From 310 × 3 onwards, apparent filling of formerly open bubble pores by lower density (darker grey) bitumen occurs, increasing from minor at 310 × 3 (Fig. 7D) to common at 350 × 3 (Fig. 7H) and decreasing at 350 × 9 (Fig. 7K). The lower density bitumen phase sometimes has clusters of dimples or small pores, giving it a spongy texture, and usually does not contain floating clay particles which are common in the lighter grey host bitumen.

##### Gordondale member

**Figure 8:**
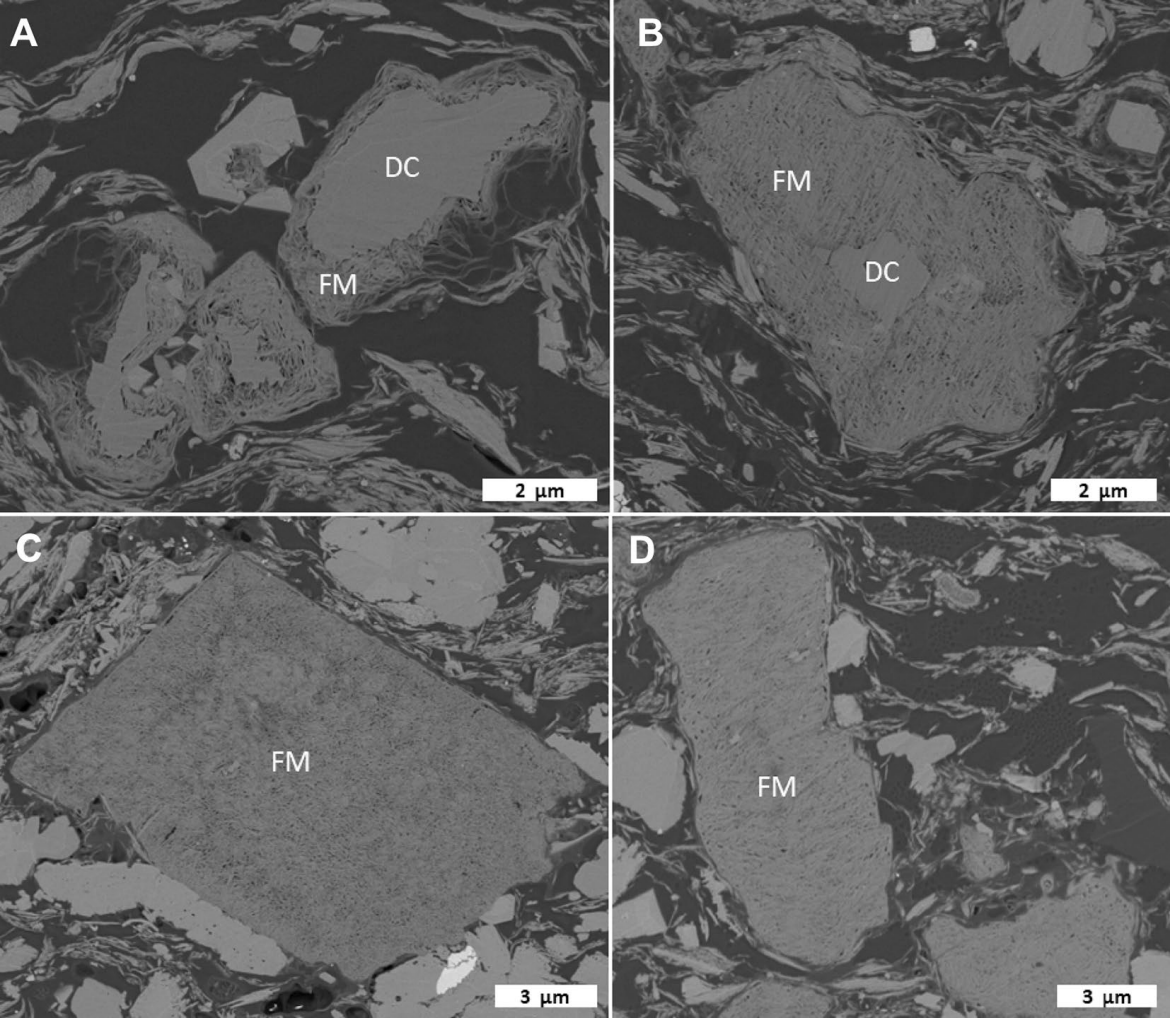
Progressive alteration of dolomite to Mg-rich fibrous masses in the GORD2 maturity series. Backscattered electron SEM images. (**A**) 310 × 3. (**B**) 340 × 3. (**C**) 350 × 3. (**D**) 350 × 9. DC: dolomite core; FM: fibrous mass.

Organic matter represents a significant proportion of the visible sample area in GORD SEM images (Fig. 5D,E). Most of the OM is bitumen, which engulfs both minerals (detrital and authigenic) and discrete OM particles (higher density, distinct edges). Observable matrix porosity is minor in immature GORD samples and is restricted to nm-scale pores in tight clusters of clay minerals. Inorganic porosity appears to increase through the maturity series, as dolomite crystals are progressively altered from rim to core into masses of fibrous Mg-rich diagenetic minerals (Fig. 8). Intergranular pores between the fibers of these masses are typically 10’s of nm in diameter but likely range from a few nm to a few hundred nm. Mg-rich masses are more common in GORD than DVRN samples.

**Figure 9:**
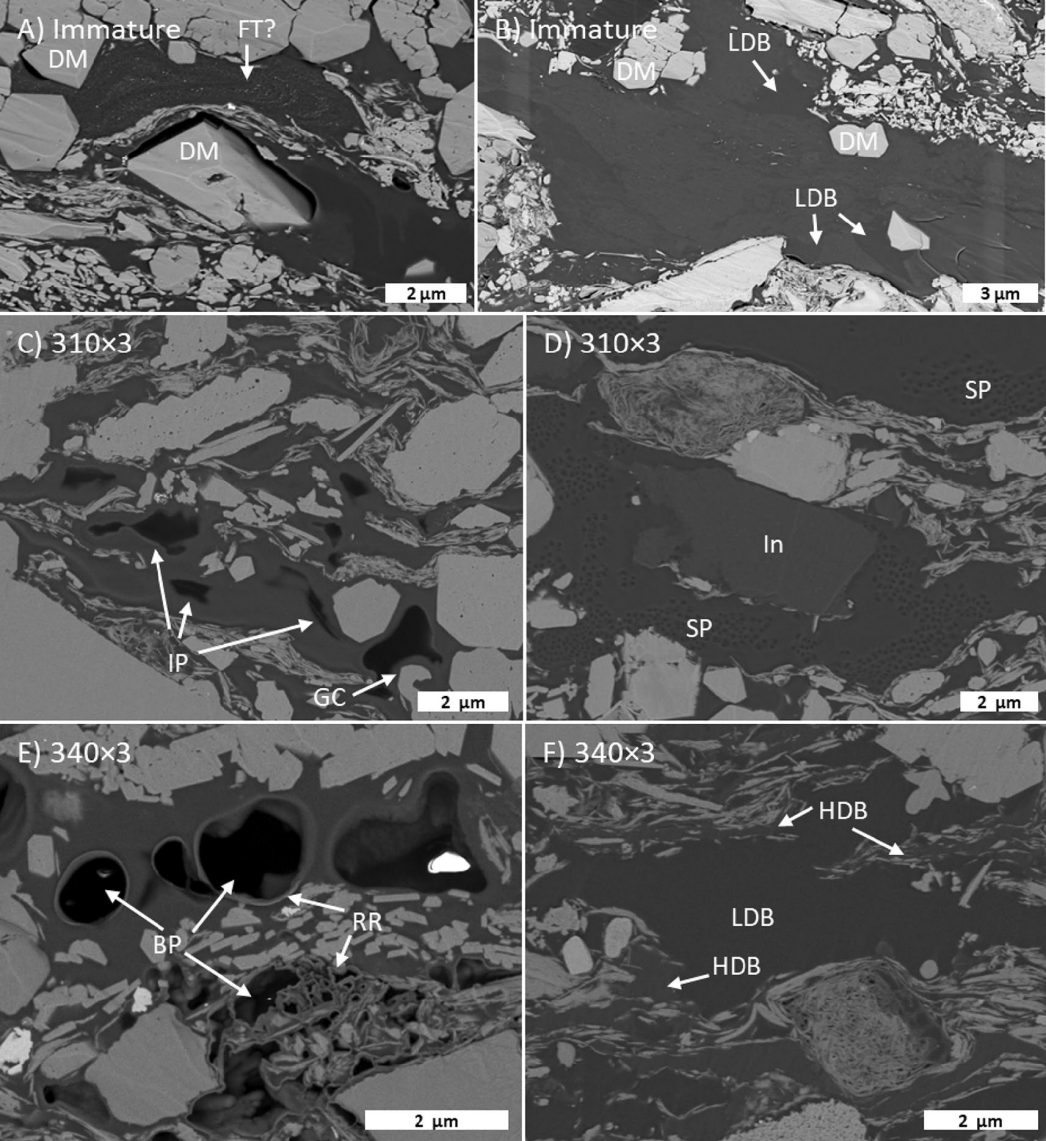

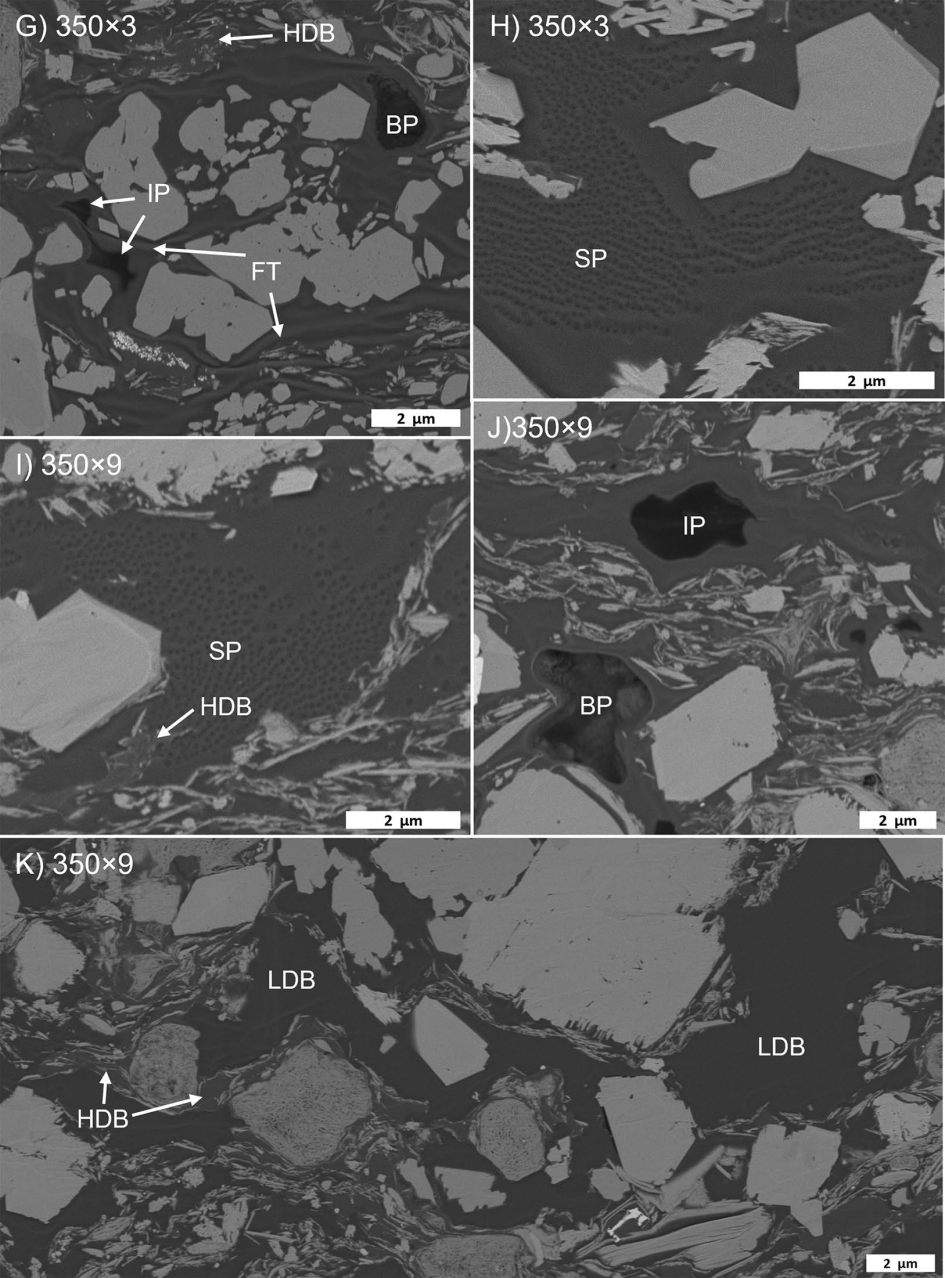
Gordondale maturity series in high-resolution (5 nm/px) backscattered electron SEM images. BP: bubble pore; IR: irregular pore; SP: spongy porosity; RR: raised rim; LDB: low-density bitumen; HDB: high-density bitumen; GC: grain coat; FT: flow textur;, In: inertinite; DM: diagenetic mineral.

Organic porosity is minor in the immature samples (Fig. 9A,B), typically as shrinkage cracks or rare primary organic porosity. Secondary organic porosity increases to the 340 × 3 stage (Fig. 9C–F) and then decreases (Fig. 9G–K). Two main secondary OM pore types were observed: (1) large (~ 1–2 µm) bubble- or irregular-shaped pores, and (2) sponge-like clusters of ~ 100–200 nm pores, often within large regions of solid bitumen. Irregular pores are often surrounded by thick (10’s-100’s nm) coats of bitumen on adjacent mineral particles (Fig. 9C,J). The pore size of each pore type group appears relatively constant throughout the maturity series. At all maturity stages, and particularly from 340 × 3 onwards, two phases of bitumen are visible based on greyscale contrast. The lower density (darker) phase contains organic porosity and fluid-like textures.

#### MICP and N_2_-adsorption

**Figure 10:**
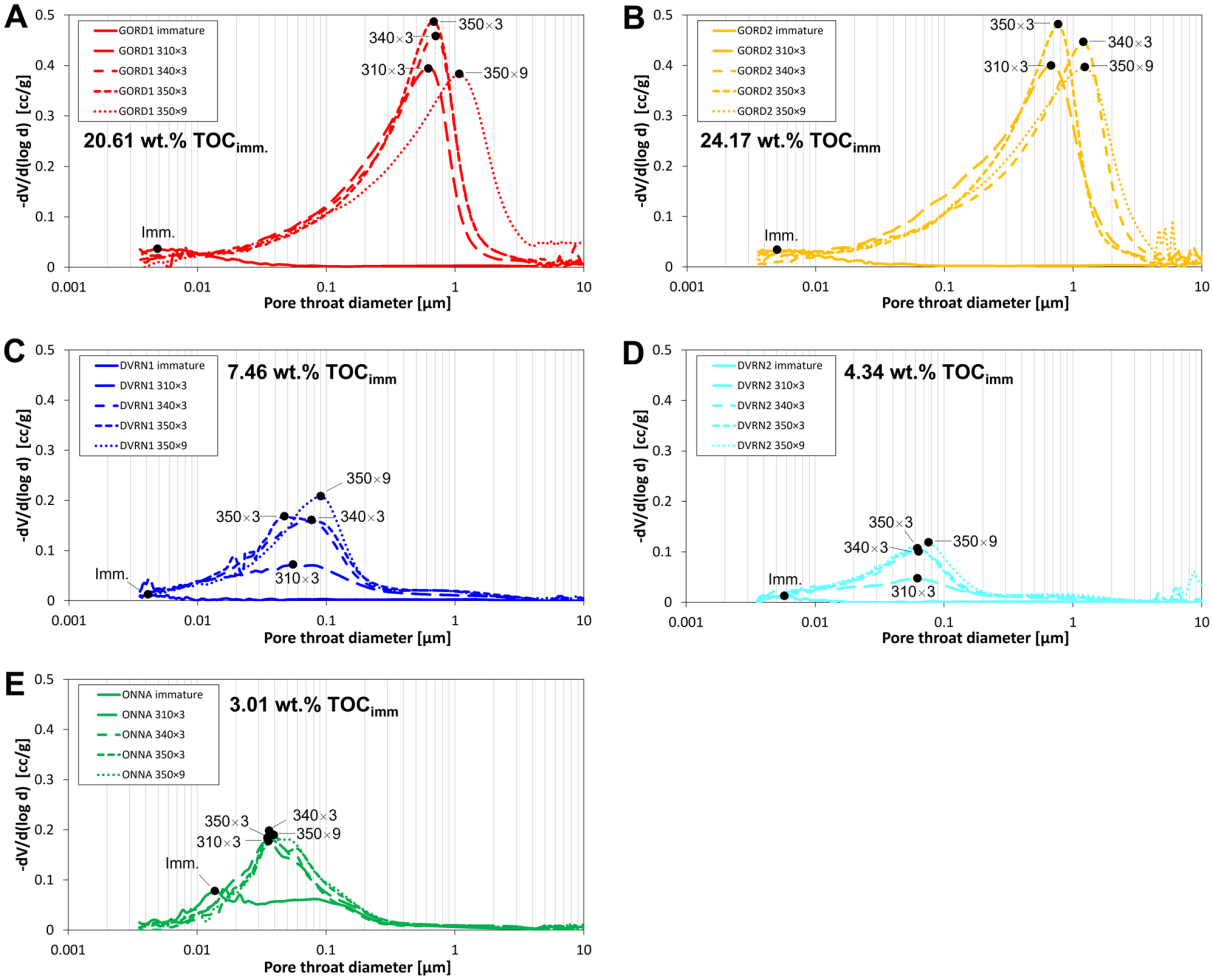
MICP pore throat size distributions for (**A**) GORD1, (**B**) GORD2, (**C**) DVRN1, (**D**) DVRN2 and (**E**) ONNA. Black circles indicate the peak pore throat diameter of each measurement. TOC_imm_: measured TOC of immature samples.

**Figure 11:**
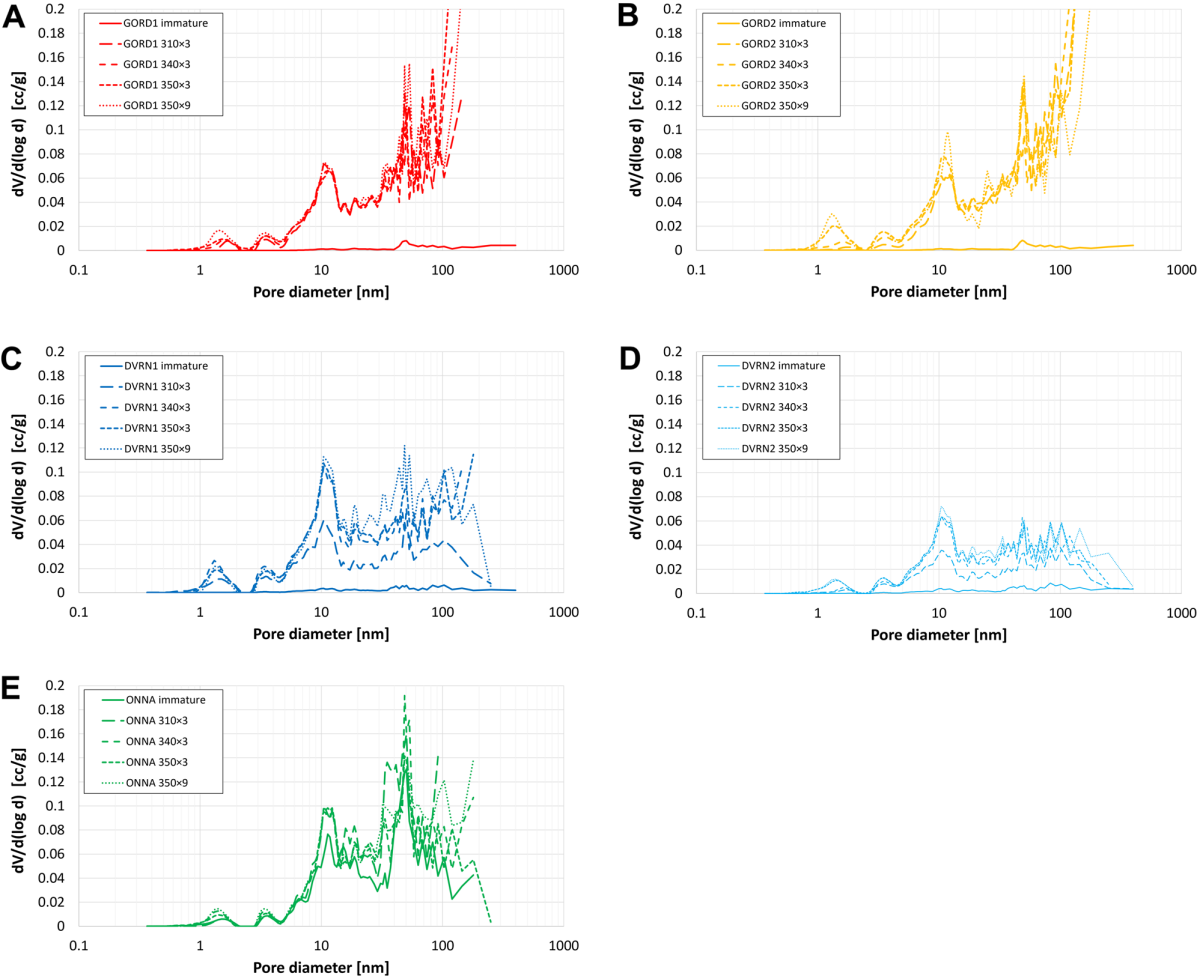
N_2_-adsorption pore size distributions for (**A**) GORD1, (**B**) GORD2, (**C**) DVRN1, (**D**) DVRN2 and (**E**) ONNA.

**Figure 12:**
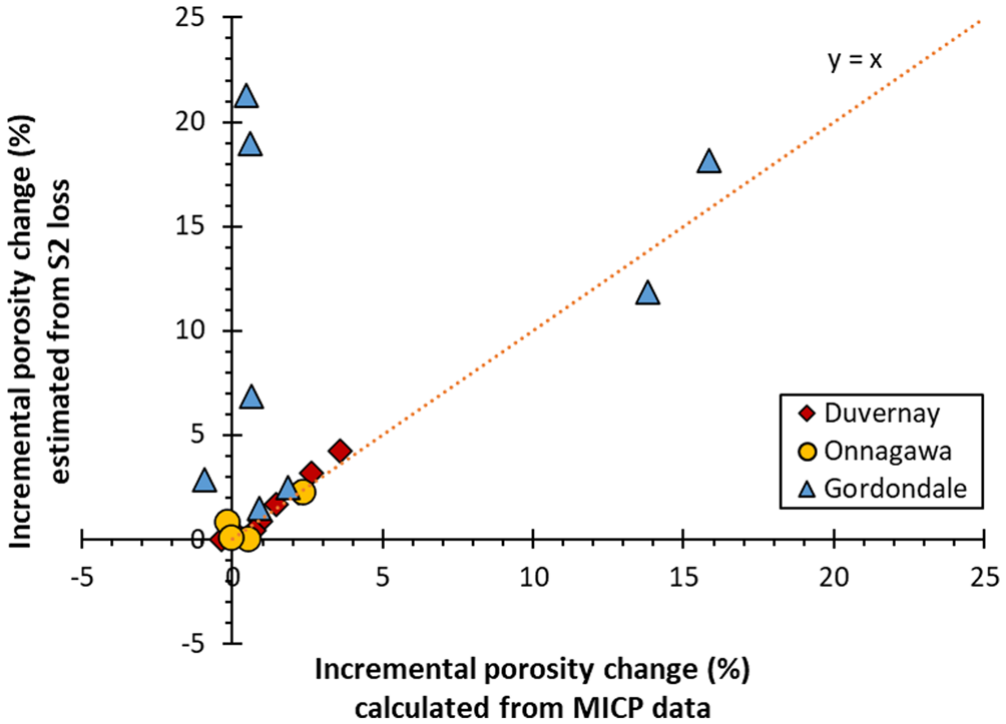
Comparison of incremental porosity change estimated from MICP and Rock–Eval S2.

The present study analyzes the matrix pore system and thus does not utilize MICP data corresponding to > 4 µm pore throat diameter to avoid intruded volume associated with conformance, artificial fractures, and experimental artefacts. MICP (Fig. 10) and N_2_-adsorption (Fig. 11) data (Table 5) show that pore volume is close to zero in immature GORD and DVRN samples, but much higher in ONNA, consistent with SEM observations that show common matrix porosity in the immature ONNA sample. Total pore volumes increase significantly in all samples in response to thermal maturation. ONNA samples have the smallest growth in pore volume, followed by DVRN, then GORD, a trend consistent with increasing TOC content. Incremental porosity changes at each successive maturity stage, as estimated from MICP are consistent with those estimated from the loss of S2 (which represents the porosity-hosting OM phases) (Fig. 12). The 4 outliers are GORD1 and GORD2 samples at 340 × 3 and 350 × 3, which show extensive low-density, pore-filling solid bitumen in SEM images. The presence of extensive low-density bitumen breaks the assumption that S2 OM density is equal to 1/3 matrix density, thus overestimating S2-loss porosity. MICP pore throat size distributions (PTSD) vary significantly between sample groups (Fig. 10). Most pore volume growth for DVRN and ONNA samples occurs in pores with pore throat diameters below about 0.3 µm, with maximum amplitudes in the range of approximately 0.03 to 0.1 µm. Minor pore volume growth occurs in the pore throat range of 0.3 to 4.0 µm. Most pore volume growth for GORD samples occurs in pores with larger pore throats, below about 3.0 µm, with maximum amplitudes in the range of approximately 0.4 to 2.0 µm. DVRN and ONNA samples generally have MICP pore throat sizes much smaller than SEM-observed pore body diameters while GORD samples have MICP pore throat diameters similar to SEM-observed pore body diameters.

**Table 5:** Measured pore volume and estimated porosity (assuming bulk density = 2.4 g/cc) from MICP and N_2_-adsorption.

Sample family	HP temperature and duration	Pore volume (cc/g)		Estimated porosity (%)	
		MICP	N_2_	MICP	N_2_
DVRN1	Immature	0.0063	0.0038	0.3	0.2
	310 °C × 3 days	0.0927	0.0487	3.9	2.0
	340 °C × 3 days	0.1553	0.0829	6.5	3.5
	350 °C × 3 days	0.1777	0.0860	7.4	3.6
	350 °C × 9 days	0.1686	0.0977	7.0	4.1
DVRN2	Immature	0.0045	0.0045	0.2	0.2
	310 °C × 3 days	0.0569	0.0303	2.4	1.3
	340 °C × 3 days	0.0919	0.0482	3.8	2.0
	350 °C × 3 days	0.0921	0.0545	3.8	2.3
	350 °C × 9 days	0.1092	0.0556	4.6	2.3
ONNA	Immature	0.0911	0.0594	3.8	2.5
	310 °C × 3 days	0.1463	0.0866	6.1	3.6
	340 °C × 3 days	0.1415	0.0887	5.9	3.7
	350 °C × 3 days	0.1536	0.0930	6.4	3.9
	350 °C × 9 days	0.1518	0.1016	6.3	4.2
GORD1	Immature	0.0141	0.0025	0.6	0.1
	310 °C × 3 days	0.3458	0.0696	14.4	2.9
	340 °C × 3 days	0.3599	0.0730	15.0	3.0
	350 °C × 3 days	0.3753	0.0793	15.6	3.3
	350 °C × 9 days	0.3965	0.0813	16.5	3.4
GORD2	Immature	0.0135	0.0022	0.6	0.1
	310 °C × 3 days	0.3942	0.0654	16.4	2.7
	340 °C × 3 days	0.4049	0.0736	16.9	3.1
	350 °C × 3 days	0.3821	0.0914	15.9	3.8
	350 °C × 9 days	0.4266	0.0822	17.8	3.4

Pore size distributions (PSD) from N_2_-adsorption (Fig. 11) are more similar across the sample groups compared to MICP PTSD. For all samples, most of the N_2_ pore volume occurs in the size range of about 5–100 nm, which is the upper pore diameter limit of N_2_-adsorption measurements, thus preventing detection of µm-scale bubble- and irregular-type pores. Differences in PSD are most notable in the size range of about 20–100 nm, roughly coincident with the SEM-observed pore size ranges of spongy porosity and fibrous mineral masses. In this range ONNA pore volumes generally decrease with increasing pore size and there is no significant change in volume through the maturity series (Fig. 11E). DVRN pore volumes moderately increase with increasing pore size and there is significant pore volume increase from low to high thermal maturity (Fig. 11C,D). GORD pore volumes steeply increase with increasing pore size and there is very significant pore volume increase from low- to high-thermal maturity (Fig. 11A,B). These observations are consistent with SEM observations that the occurrences of spongy porosity and porous fibrous mineral masses increase with increasing thermal maturity and are non-existent, minor/moderate, and abundant in ONNA, DVRN, and GORD, respectively. Peak pore throat diameter (PPTD), the maximum amplitude of the PTSD increases significantly in all five sample groups from immature to 310 × 3 (Fig. 10), after which PPTD increases only slightly and discontinuously towards maximum thermal maturity. PPTD decreases from 340 × 3 to 350 × 3 in DVRN and GORD samples.

**Figure 13:**
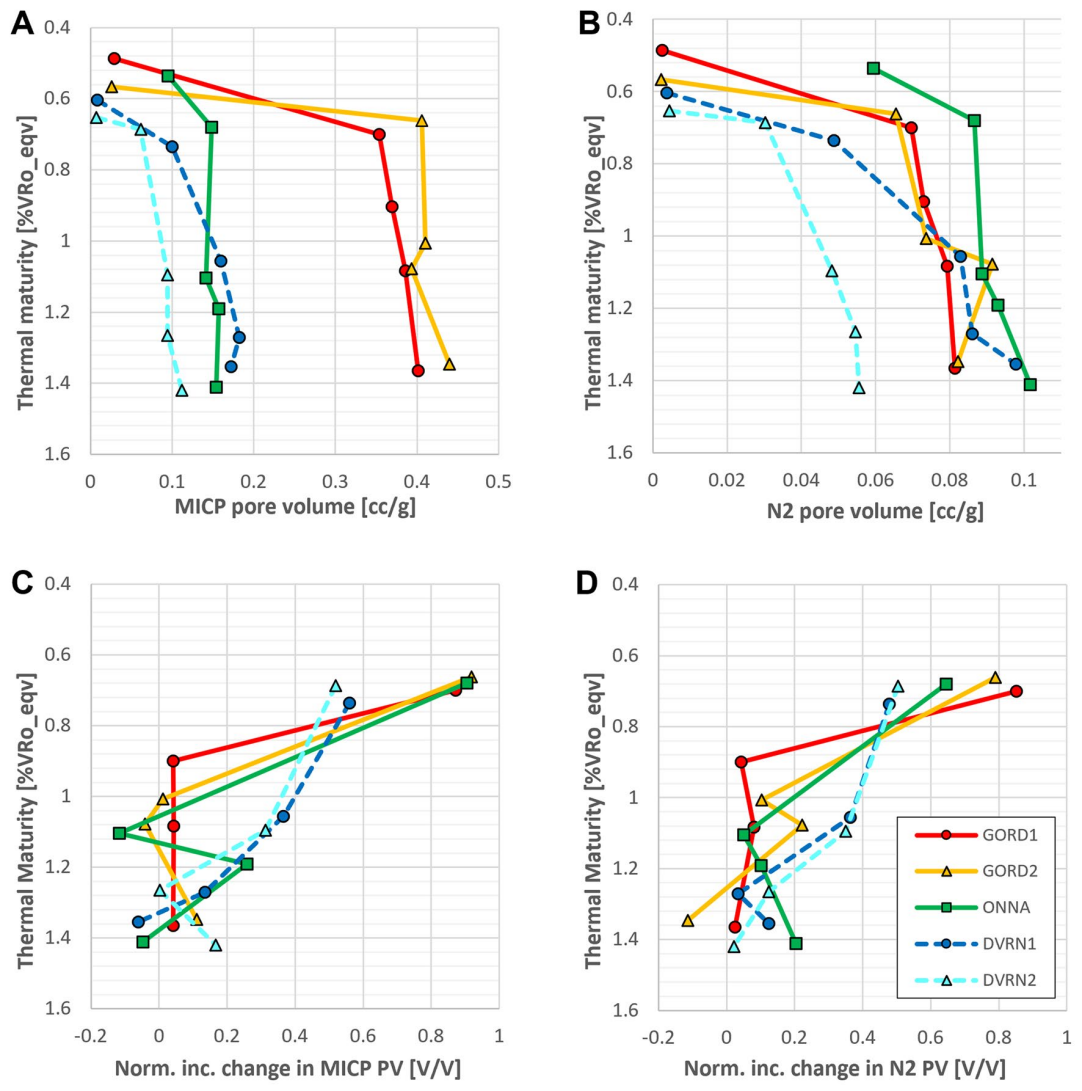
(**A**, **B**) Intruded pore volume from MICP (**A**) and N_2_-adsorption (**B**) vs thermal maturity. Maturity increases down the y-axis as a proxy for burial depth. (**C**, **D**) Normalized incremental change in MICP (**C**) and N_2_ (**D**) pore volume vs. thermal maturity. Normalized incremental PV change = (PV_n_ − PV_n−1_)/(PV_350×9_ − PV_immature_). Total porosity change from immature to maximum maturity was normalized to 1. Each data point represents the fraction of the total pore volume change that occurred at each maturity stage, and as such, there is no data for immature samples.

The timing of pore volume growth is variable across sample sets. In Type IIS samples (GORD and ONNA), approximately 90% of the total change in MICP-detected pore volume occurs from immature to 310 × 3, and subsequent porosity change is very minor (Fig. 13A,C). Similarly, 65–85% of the total N_2_ pore volume generation (Fig. 13B,D), occurs by 310 × 3 in GORD and ONNA samples. In contrast, only about 45–55% of the total MICP (Fig. 13C) and N_2_ (Fig. 13D) pore volume change had occurred by 310 × 3 for the low-TOS DVRN samples, while another 30–40% occurred between 310 × 3 and 340 × 3. In terms of VRo_eqv_, GORD and ONNA samples exhibit the most drastic pore volume generation below about 0.70% while DVRN samples have more evenly distributed pore volume generation up to approximately 1.1% VRo_eqv_.

## Discussion

### Porosity origins

Organic pores were generated in all samples during HP thermal maturation. Organic pores were observed directly in SEM images and significant pore volume increases were detected by MICP and N_2_-adsorption measurements (Figs. 11, 12). The magnitude of pore volume change from immature to the final HP stage was lowest in ONNA, followed by DVRN, then GORD sample sets, consistent with increasing TOC content, suggesting pore volume growth is primarily associated with organic pores. Furthermore, incremental porosity change estimated by S2 loss is similar to estimations from MICP, indicating that pore volume change is dominated by S2 OM (Fig. 12). These observations are consistent with a large body of published research showing that thermal maturity is a first order control on organic porosity generation^11–16,48,49^. However, this study also demonstrates the generation of *inorganic* pores with increasing thermal maturity—specifically dolomite alteration to Mg-rich fibrous masses. Positive correlation between pore volume and thermal maturity in organic-rich rocks cannot always be completely attributed to organic porosity (e.g.,^50–52^). More research is warranted to understand the occurrence of similar dolomite alteration in natural settings.

### Porosity destruction

There are many processes that can reduce pore volume, but these processes can be generalized as either compaction or occlusion. Compaction is not relevant to our samples as they were artificially matured without overburden load, but occlusion may be significant, particularly by oil and bitumen (i.e., migrabitumen *sensu*^42^), which can then be transformed into solid bitumen or pyrobitumen^14,16,19–21,23,53^ across a wide thermal maturity range^54–56^ (among others). The presence of pore-filling solid bitumen has been documented across our HP data set over a maturity range consistent with that observed in other natural^14,16,19–21,23,53^ and artificial settings^57–59^ Pore-filling textures are apparent in SEM (Figs. 7, 9) and organic petrographic images (Fig. 5) of all GORD and DVRN samples (0.49–1.42% VRo_eqv_). Furthermore, at least two phases of pore-filling solid bitumen were observed in GORD samples, based on greyscale contrast in SEM images, bimodal bitumen reflectance populations, and an interpretation that outliers in a cross plot of MICP vs S2-loss porosity (Fig. 12) correspond to the generation of a voluminous low-density, pore-filling solid bitumen phase. The lower density phase of solid bitumen can sometimes be observed at low maturity but becomes much more prevalent from 340 × 3 onwards (greater than about 0.9% VRo_eqv_) and seems to fill previously generated organic pores (e.g., Fig. 7G,H,K). These observations support the notion that the composition of pore-filling fluids (that later transform to solid bitumen) evolve with thermal maturity, as observed in laboratory^55–59^ and natural settings^20,57,58^. In this case, the later solid bitumen phase is less dense. The observations additionally demonstrate that pore-occlusion by solid bitumen is not restricted to inorganic pores.

### Timing of organic porosity generation and destruction

The central hypothesis of this study was that organic porosity should be generated sooner (at lower thermal maturity) in samples with high TOS content (i.e., Type IIS kerogen) as source rocks with Type IIS kerogen are known to generate oil earlier than those with low TOS content (e.g., Type II kerogen)^33–39^. The data from this study positively support the hypothesis, and we believe this work is the first published demonstration of this phenomenon. Figure 12 illustrates that in Type IIS samples (GORD and ONNA), the large majority of total pore volume increase occurs below about 0.70% VRo_eqv_. In contrast, pore volume increase in the low-TOS DVRN samples is more broadly distributed, and robust pore volume growth continues until approximately 1.1% VRo_eqv_, with subsequent minor increase to about 1.4% VRo_eqv_ above which we do not have data. Prolonged pore volume growth is common in artificially matured organic-rich shales with low TOS concentrations^60–64^. The contrasting pore-volume trends between Type II and Type IIS samples presented here suggest that organic porosity generation occurs earlier in samples with high TOS relative to those with low TOS. These observations are evident despite the significant variation in TOC content, mineralogy, and texture between sample sets. GORD samples have drastically higher TOC values than ONNA samples, and ONNA samples have a tight microcrystalline quartz matrix drastically different from the matrix and mineralogy of GORD samples, but both sample sets exhibit a strong dominance of pore volume increase at low thermal maturity relative to the DVRN sample sets. TOS content influences porosity generation timing but not magnitude of change, which is controlled by TOC.

Organic porosity generation reported here, between ~ 0.5–1.4%VR_eqv_ is consistent with myriad studies showing secondary organic pore generation in natural systems in this maturity range^28,29^ (among others), tempered by compaction, which is absent here, and occlusion by solid bitumen. Organic porosity generation is also known to occur at higher thermal maturity but was not tested in this study given the maximum maturity of ~ 1.4% VR_eqv_. No study known to the authors has examined organic pore system evolution as a function of TOS content in natural systems. The present experimental study lays the groundwork for such research.

TOS content influences the timing of hydrocarbon and porosity generation and thus may also influence the timing of porosity destruction via pore-filling solid bitumen. Figure 12A and C show that the first occurrence of negative MICP pore volume change occurs at lower thermal maturity in Type IIS samples (1.08 and 1.10% VRo_eqv_) relative to Type II samples (1.35% VRo_eqv_). However, negative pore volume changes are minor and may just represent natural sample variation. The combined SEM and pore volume data suggest that porosity destruction by pore-filling solid bitumen may occur earlier in shales with Type IIS OM relative to those with Type II OM, but more investigation is warranted for a confident conclusion.

### Pore size distribution (PSD)

The PSD in artificially matured samples without overburden load, such as in the present study, should not be considered representative of *in-situ* rock properties. Compaction radically reduces pore volume at burial depths of 10 s to 100 s of meters in natural settings because water initially comprises up to 90% of the bulk volume of freshly deposited muds^3^. Furthermore, compaction-induced pore volume destruction is not limited to shallow burial or inorganic pore systems; organic porosity, at thousands of meters burial depth is also subject to compaction by overburden load or tectonic stress^28,29^, particularly when volumes of rigid matrix minerals are low. These processes, in addition to pore-filling by bitumen, are responsible for the extreme low pore volumes of immature samples reported here but had no influence on pores generated during hydrous pyrolysis.

That caveat aside, there is still considerable insight to be gained from PSD and pore types observed in artificially matured samples, particularly the relationships between pore body size, pore throat size, and rock fabric. Across samples, it is commonly observed that the large organic pores (i.e., bubble- and irregular-shaped) have sizes limited by adjacent minerals. Rock fabric—specifically the distances between minerals or inert OM—controls maximum organic pore body size and defines the size of pore throats through which penetrating fluids (including Hg) must pass to access organic pores. Large organic pores observed in SEM are typically several hundred nanometers to a few micrometers across in all samples, but MICP PTSD are highly variable between sample sets, and skewed to lower diameters in samples with restrictive matrix fabrics and discontinuous OM particles (i.e., DVRN and ONNA). Naturally-matured organic-rich mudstones likely also experience the phenomenon of rock fabric limiting access to organic pores unless OM is highly voluminous and continuous.

## Conclusions

This study examined the influence of total organic sulfur (TOS) content on the generation of bitumen and secondary organic porosity in five sample groups of organic-rich mudstones from the Duvernay Formation (Canada), Onnagawa Formation (Japan), and Gordondale member of the Fernie Formation (Canada). Pore volume increase with thermal maturity was seen in all five sample groups, with the magnitude of pore volume generation increasing with increasing TOC. SEM observations confirmed that most pores generated during HP were organic pores. However, inorganic porosity, linked to the progressive alteration of dolomite to fibrous masses, a relatively novel observation, was illustrated as evidence that correlations between porosity and thermal maturity in organic-rich rocks cannot always be completely ascribed to organic porosity. The size ranges of organic pores were relatively constant across samples and thermal maturity, which was interpreted to be the result of the absence of compressive forces during thermal maturation. MICP PTSD was controlled by rock fabric, particularly the spacing between mineral grains, which constricted flow of Hg into organic pore networks.

Evaluations of the pore space using SEM, MICP, and N_2_-adsorption support the hypothesis that organic porosity is created at lower thermal maturity in marine kerogen of Type IIS relative to Type II. This conclusion fits well with the rich history of literature that demonstrates early generation of liquid hydrocarbons in Type IIS source rocks and the present study is, to our knowledge, the first published demonstration of this phenomenon. This study is also novel in its illustration of solid bitumen occlusion of organic porosity, not only inorganic matrix porosity, which has not been well documented previously. Additionally, two distinct phases of solid bitumen generation were observed in SEM images and BRo measurements.

## Methodology

### Sample selection and workflow

Five thermally immature core samples were selected from three organic-rich mudstone units, which in order of increasing TOS content were the late Devonian Duvernay Formation (Canada), middle-late Miocene Onnagawa Formation (Japan), and early Jurassic Gordondale (formerly Nordegg) member of the Fernie Formation (Canada). Duvernay Formation mudstones are primarily calcareous to siliceous, as a function of high biogenic silica production and the deposition of reef- and shelf-derived fine-grained carbonate sediment deposited in an epicontinental seaway on the flooded passive margin of western North America^65–69^. The Onnagawa Formation is composed of siliceous, diatomaceous mudstones and siltstones that were deposited in deep, silled sub-basins in the paleo-Sea of Japan, under highly bioproductive surface waters driven by intense upwelling^70–72^. The Gordondale member is composed of organic-rich, phosphatic and highly radioactive calcareous-siliceous mudstones and fine-grained calcarenites^73,74^. that were deposited in an epicontinental seaway that occupied a subsiding foreland basin along the western Canadian Cordilleran margin^75^.

Two Duvernay core samples were selected from the Long Run DD Gvillee 4–34–77–23 well at present-day burial depths of 2413.15 m and 2414.00 m. One Onnagawa core sample was selected at a depth of 16.57 m from a shallow science well in Akita Prefecture. Two Gordondale core samples were selected from the Adamant Berwyn 11–32–82–25 well at depths of 924.70 m and 926.00 m.

**Figure 14:**
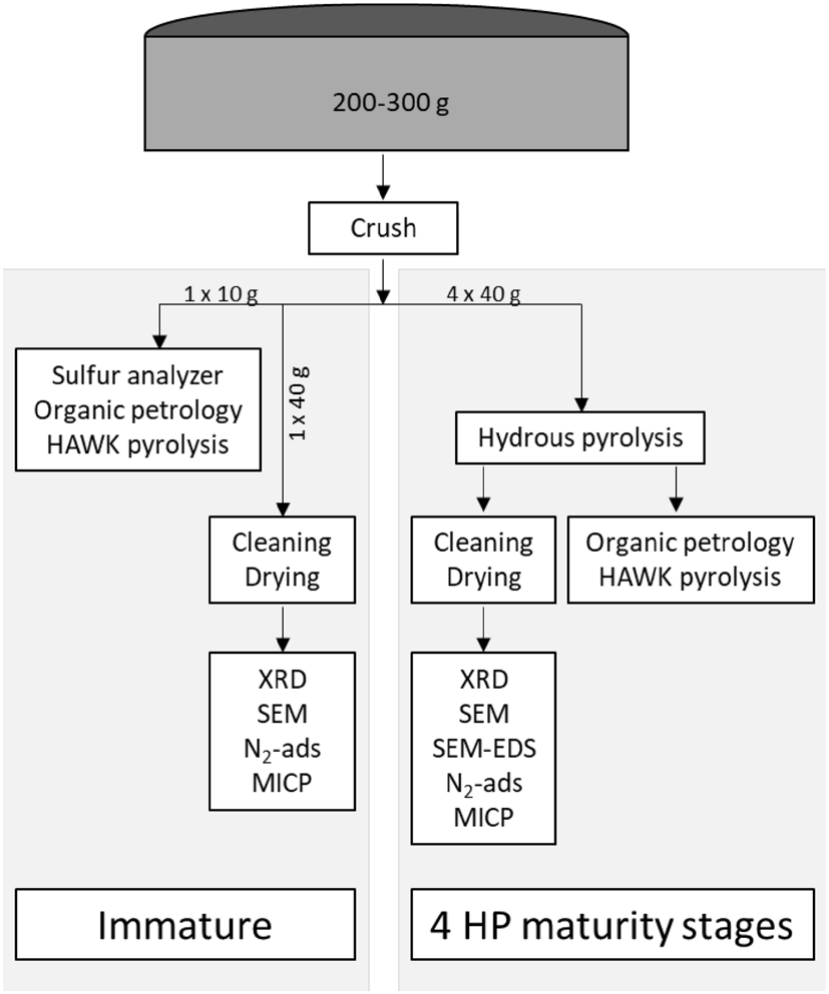
Workflow for sample processing and experimentation.

Each of the five core samples consisted of approximately 200 g of continuous core slab, representing 15–25 cm of vertical thickness (Fig. 14). Each sample was crushed to < 2.0 mm and homogenized then separated into 5 aliquots. Organic geochemical, mineralogical, petrophysical, and high-resolution imaging experiments were performed on thermally immature sample aliquots and on 4 additional aliquots of equivalent sample material at matured to successively higher thermal maturity stages through hydrous pyrolysis. A total of 25 unique samples were analyzed (5 core samples × 5 maturity stages) using the methods of organic petrology, HAWK programmed pyrolysis, X-ray diffraction (XRD), scanning electron microscopy (SEM), low pressure N_2_ gas adsorption, and mercury injection capillary pressure (MICP). Sulfur analysis using Rock-Eval 7S was performed on only the immature sample aliquots. Elemental concentration maps of high-maturity samples were generated using energy-dispersive X-ray spectroscopy (SEM–EDS). Prior to mineralogical, textural, and petrophysical measurements, crushed sample material was cleaned using Dean Stark–Soxhlet extraction to remove soluble bitumen and lighter hydrocarbons.

### Hydrous pyrolysis

Hydrous pyrolysis (HP) was performed to simulate *in-situ* thermal maturation using a 500 ml Parr reactor equipped with a pressure gauge. For each HP run, 40 g of homogenized crushed rock (2 mm particle size) and 100 ml distilled water were placed inside the reactor. The reactor was sealed and pressurized at 100 psi using inert argon gas. Separate HP runs were performed at isothermal temperatures of 310, 340, and 350 °C for 3 days and 350 °C for 9 days. Based on previous HP studies, the selected temperature range was within the simulated thermogenic hydrocarbon generation and early gas window^33,36,76–79^. After each run, the produced gas, oil, and rocks were collected separately and prepared for further analyses. The peak pressure ranged from 1475 psi = 100 atm @ 310 °C and 2675 psi = 182 atm @ 350 °C.

### Total sulfur speciation

Approximately ~ 50 mg of bulk powder was analyzed using a Rock–Eval 7S analyzer (Vinci Technologies, France). The sulfur speciation analysis was performed using the basic/total sulfur method^80^. The temperature program was similar to the basic programmed pyrolysis method^81^. It started at a 300 °C iso-temperature for 3 min, followed by a 25 °C/min ramp to 652 °C, but with an extra oven step during the pyrolysis stage, which is a sulfur oven where the evolved gases are oxidized into SO_2_ at 840 °C. Samples were then transferred to an oxidation oven with an extended analysis time during the oxidation stage and a 20 °C/min ramp from 300 to 1200 °C for decomposition of sulfate moieties^80,82^. The SO_2_ gas released during both pyrolysis and oxidation stages was measured in real time by an ultraviolet (UV) detector. In addition to detection of total sulfur (TS) and total organic sulfur (TOS), the instrument separated and quantified pyritic sulfur (Fe-S), bitumen/oil organic sulfur (S1-S), organic sulfur associated with hydrocarbon prone kerogen (S2-S), and residual organic sulfur associated with oxidized OM (ROS or S4-S) and sulfate minerals. The basic programmed pyrolysis parameters such as S1, S2, S3, Tmax, and TOC were measured during the same process following the Behar et al. methodology^81^.

### Basic programmed pyrolysis

Bulk sediment samples (~ 70 mg) were analyzed by HAWK (Wildcat Technologies) programmed pyrolysis. The pyrolysis stage (under an N_2_ environment) involved the initial iso-temperature of 300 °C for 3 min to release free hydrocarbons in the samples (S1, mg HC/g rock), followed by ramping the temperature up 25 °C/min to 650 °C to release, through thermal cracking, hydrocarbons and the oxygen contained in pyrolizable kerogen (S2, mg HC/g rock, and S3, mg CO_2_/g rock, respectively). Samples were then automatically transferred to the oxidation oven and heated from 300 to 850 °C at a heating rate of 20 °C/min to measure the residual inert organic carbon (S4, mg CO, and CO_2_/g rock and residual carbon (RC) wt%) and a portion of the mineral carbon (MinC, wt%).

Total organic carbon (TOC, wt%) was quantified as the sum of the total quantity of organic matter released during pyrolysis (pyrolizable carbon, PC wt%) and the oxidation step (residual carbon, RC wt.%). The oxygen index (OI) was calculated by normalizing the quantity of the pyrolizable CO_2_ (S3) to total organic carbon (S3/TOC × 100) and is proportional to the elemental O/C ratio of the kerogen while the hydrogen index (HI) is the ratio of (S2/TOC × 100) and is proportional to H/C^81^.

### Organic petrology

Organic petrography was carried out on selected samples using polished blocks made with a cold-setting epoxy–resin mixture. The resulting sample pellets were ground and polished, in final preparation for microscopy, performed using an incident light Zeiss Axioimager II microscope system equipped with an ultraviolet (UV) light source and the Diskus-Fossil system. Fluorescence microscopy of organic matter was carried out using UV G 365 nm excitation with a 420 nm barrier filter. Random reflectance measurements were conducted under oil immersion (objective × 50) following ASTM methodology^83^. The standard reference for reflectance measurement was yttrium–aluminum-garnet with a standard reflectance of 0.906% under oil immersion.

### Scanning electron microscopy and energy dispersive X-ray spectroscopy

Prior to mineralogical, textural, and petrophysical measurements, sample material was cleaned using Dean Stark–Soxhlet extraction. Toluene was percolated through the samples for three days and the produced water volumes were recorded in a graduated sidearm collection tube. Subsequently, samples were placed in effluent methyl alcohol (methanol) for one day and then in a methanol/chloroform (83% / 17%) azeotrope for 3 days. The samples were dried in a vacuum oven at 80 °C until daily weight measurements stabilized.

For scanning electron microscopy (SEM) approximately 0.5 g of crushed rock material was placed into shallow boreholes within cured epoxy pellets then epoxied in place. Pellets were slowly exposed to vacuum conditions for 20–30 s to conform the epoxy to the rock particle boundaries and prevent particles being dislodged during polishing. The epoxy in the pellets was allowed to cure over 24 h without heating. Pellets were trimmed to approximately 1 × 1 × 1 cm then ground and pre-polished using a rotating abrasive wheel with fine silicon carbide sandpaper. Samples were then placed into a Fischione 1060 SEM Mill and the polished surface was ion milled using a broad argon ion beam (BIB) at low angle (relative to the sample surface). A very light carbon coating was sputtered onto the polished sample surface to reduce charging during SEM imaging. Imaging of polished surfaces was performed using a Helios NanoLab™ 650 Dual Beam™ microscope with an accelerating voltage of 2 kV and a probe current of 100 pA. All images are backscattered electron images. Twenty to 25 rock particles per pellet were imaged at 115 nm/px (“Overview”) to screen for desirable bedding orientation, texture, surface quality, and organic richness. High-resolution (5 nm/px) imaging was performed on a 114 × 114 µm area of one particle in each pellet.“Overview” and “high-res” images are mosaics of multiple stitched image tiles; stitching expands the field of view while maintaining spatial resolution.

Greyscale variation in the backscattered electron SEM images relates to average atomic mass, but it is also proportional to density of the OM because low density OM has a low C:H atomic ratio, i.e. more low-mass H atoms.

On the SEM pellets of the 350 × 9 samples, at the same locations as the high-resolution images, elemental concentration maps with a spatial resolution of 5 nm/px were generated using scanning electron microscopy with energy-dispersive X-ray spectroscopy (SEM–EDS). The instrument was a Helios NanoLab™ 650 Dual Beam™ SEM microscope with an EDS device. Data was collected with an accelerating voltage of 15 kV and a probe current of 0.8 nA.

### Mercury injection capillary pressure and N_2_-adsorption

Mercury injection capillary pressure (MICP) analysis was performed on 1 to 2 g of crushed rock using a Quantachrome Instruments PoreMaster 60GT. The measurement consisted of low-pressure analysis up to 50 psi and high-pressure analysis up to 60,000 psi for pore throat size distribution and pore volume estimation to a lower pore throat diameter limit of 3.6 nm.

Low pressure N_2_ gas adsorption analysis was performed on approximately 1 g of crushed rock using a Microtrac MRB BELSORP MAX II instrument. Before analysis, samples were heated at 100 °C for at least 4 h under diminished pressure. Measurement temperatures were kept to 77 K. Specific surface area and pore volume were evaluated using BET analysis. Pore size distribution was simulated by grand canonical Monte Carlo (GCMC).

## Supplementary Information

Supplementary Figures.

## Data Availability

In addition to data tables and figures presented in the manuscript, variations of programmed pyrolysis parameters over the HP series of all samples are shown in Fig. S1 online. Fig. S2 contains SEM–EDS images confirming the Mg-rich composition of fibrous masses.
