# Supplementary material for: Early porosity generation in organic-sulfur-rich mudstones
Source: Sci Rep. 2023 Jun 19;13:9904. doi: 10.1038/s41598-023-35259-5 (PMC10279667; doi:10.1038/s41598-023-35259-5)
Supplement: Supplementary file 1 — Supplementary Figures. [file 41598_2023_35259_MOESM1_ESM.pdf]

Supplementary Information for…

Early porosity generation in organic-sulfur-rich mudstones

Levi J. Knapp[1*], Omid H. Ardakani[2,3], Julito Reyes[2], Kazuaki Ishikawa[1]

[1] Japan Oil, Gas and Metals National Corporation (JOGMEC), Chiba, Japan
[2] Natural Resources Canada, Geological Survey of Canada, Calgary, Canada
[3] Department of Geoscience, University of Calgary, Calgary, Canada
[*] Now at Alberta Energy Regulator, Edmonton, Canada

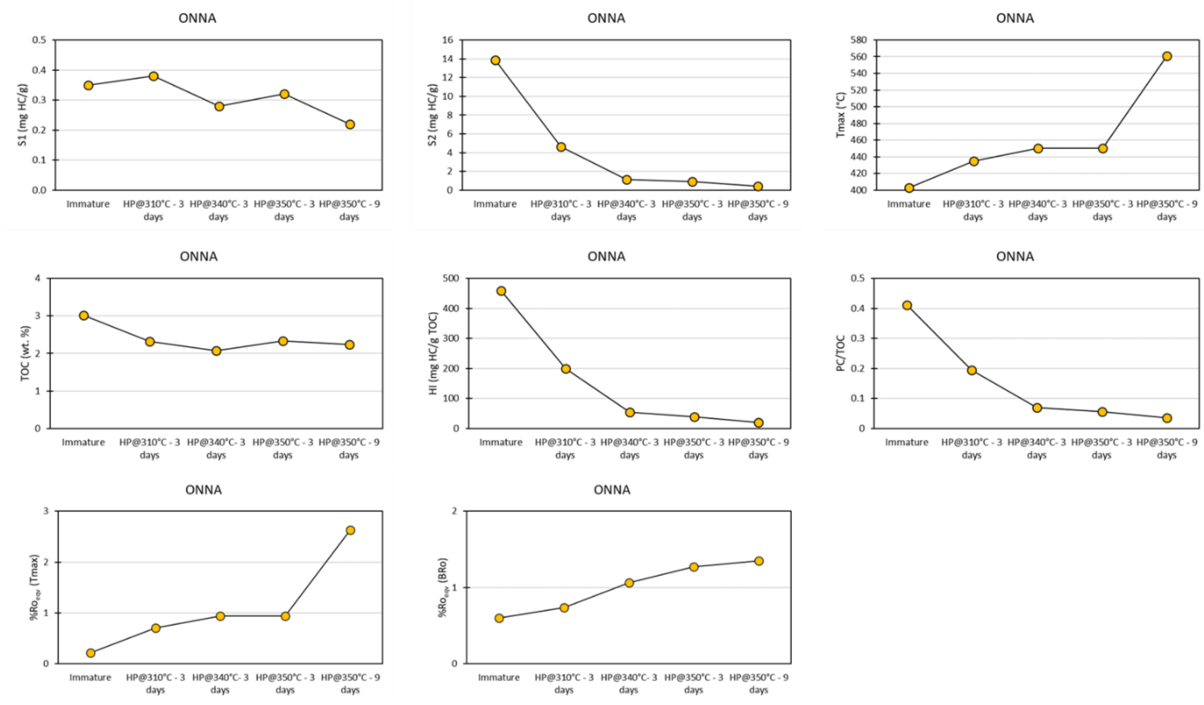

Figure S1a. Variations of programmed pyrolysis parameters over the HP series of the ONNA sample family.

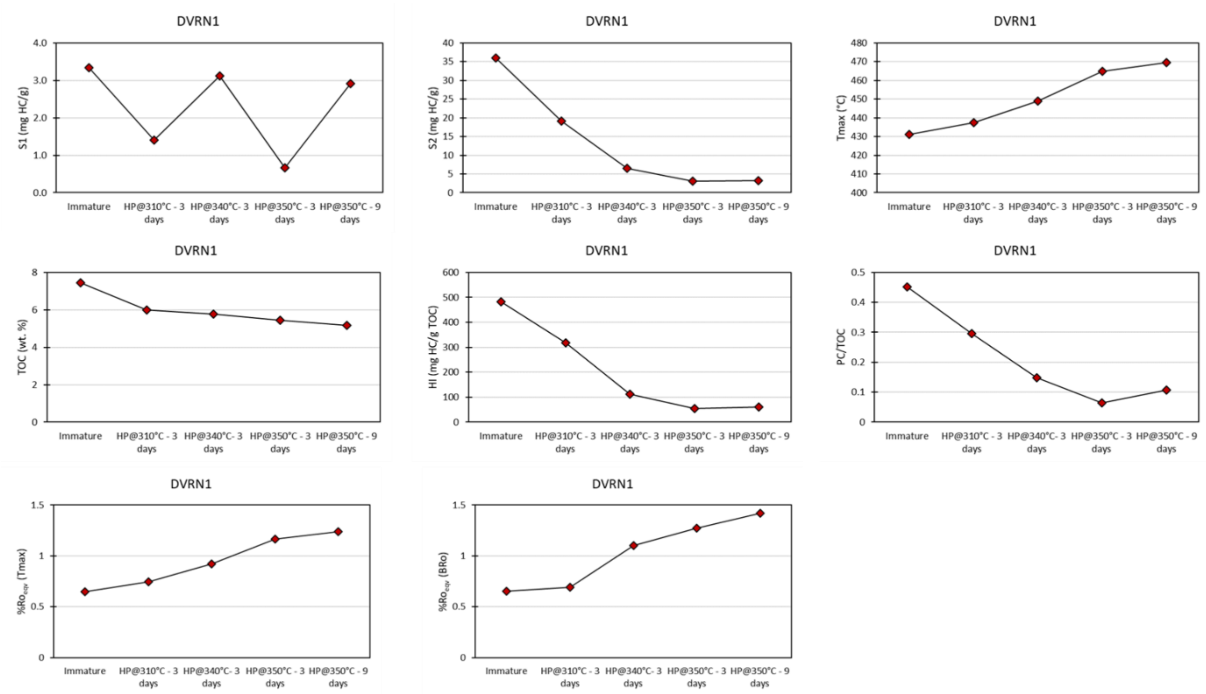

Figure S1b. Variations of programmed pyrolysis parameters over the HP series of the DVRN1 sample family.

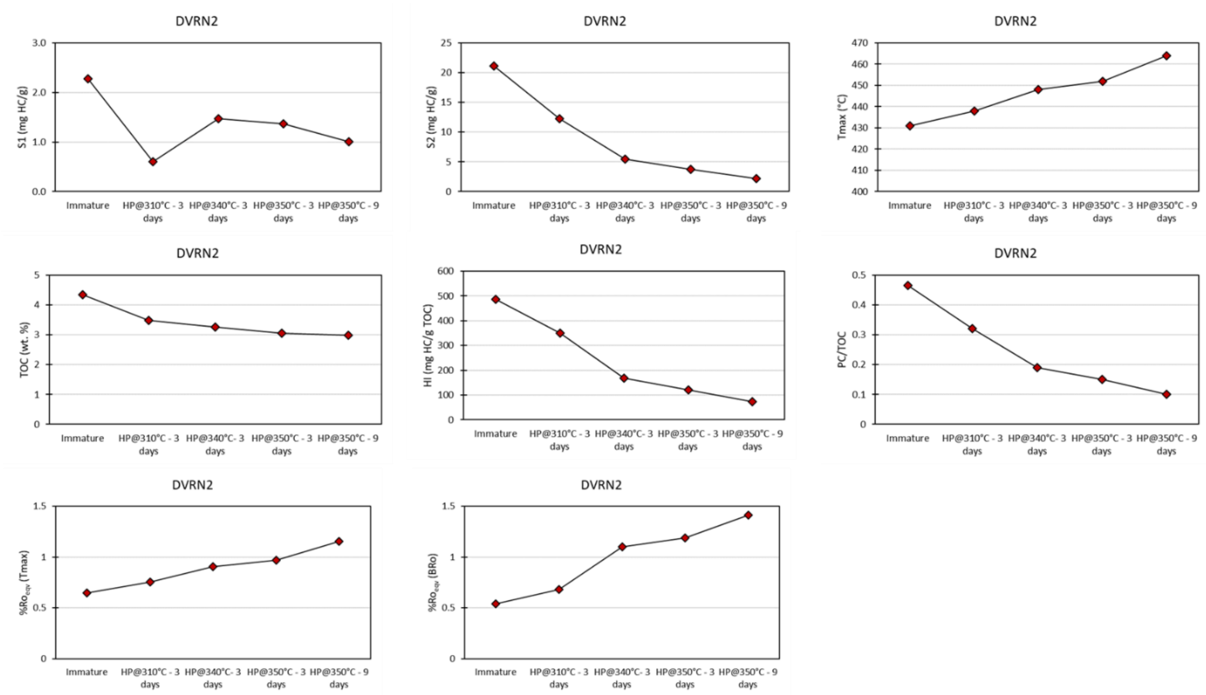

Figure S1c. Variations of programmed pyrolysis parameters over the HP series of the DVRN2 sample family.

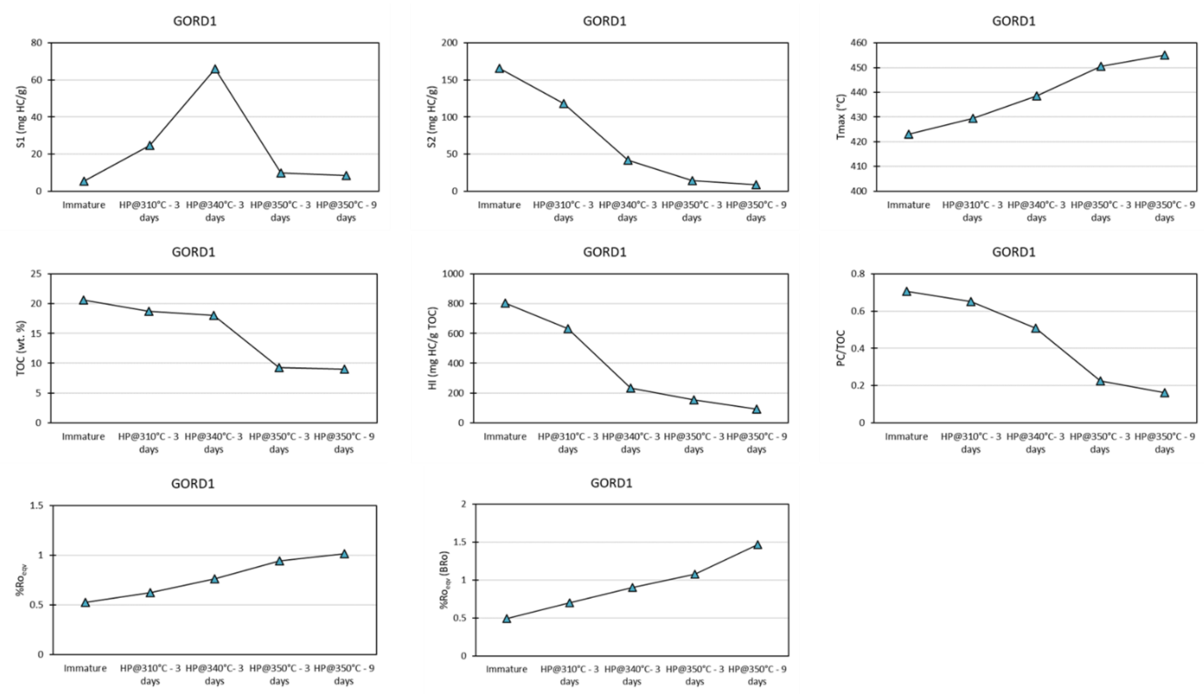

Figure S1d. Variations of programmed pyrolysis parameters over the HP series of the GORD1 sample family.

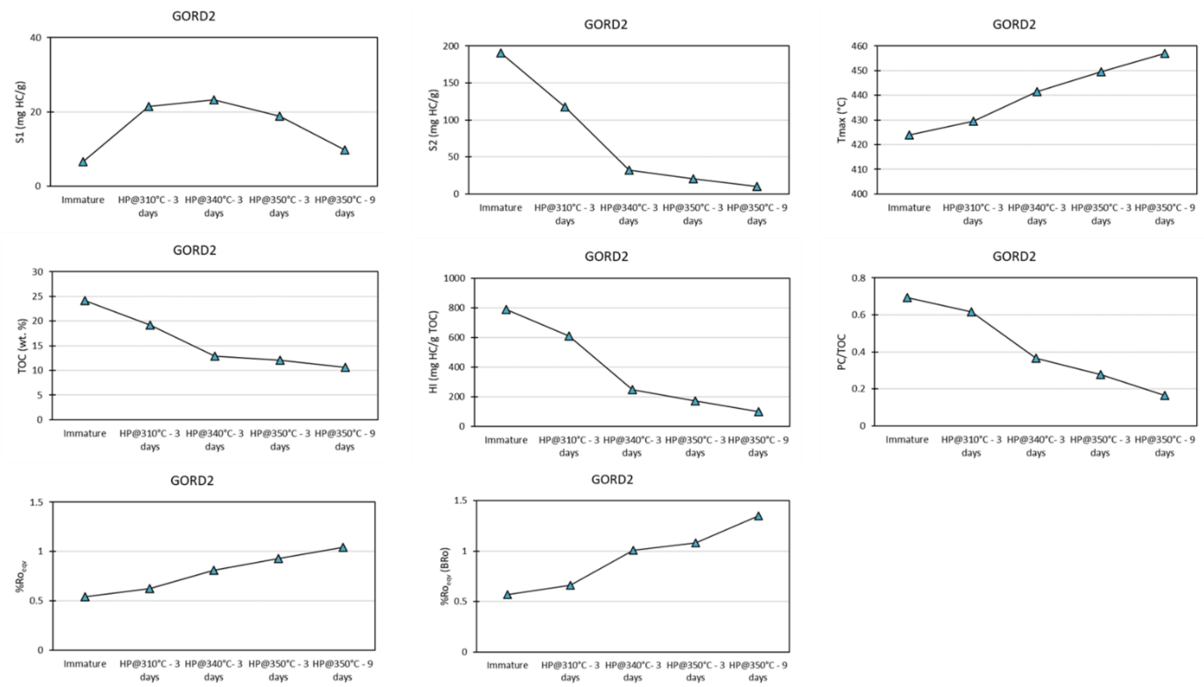

Figure S1e. Variations of programmed pyrolysis parameters over the HP series of the GORD2 sample family.

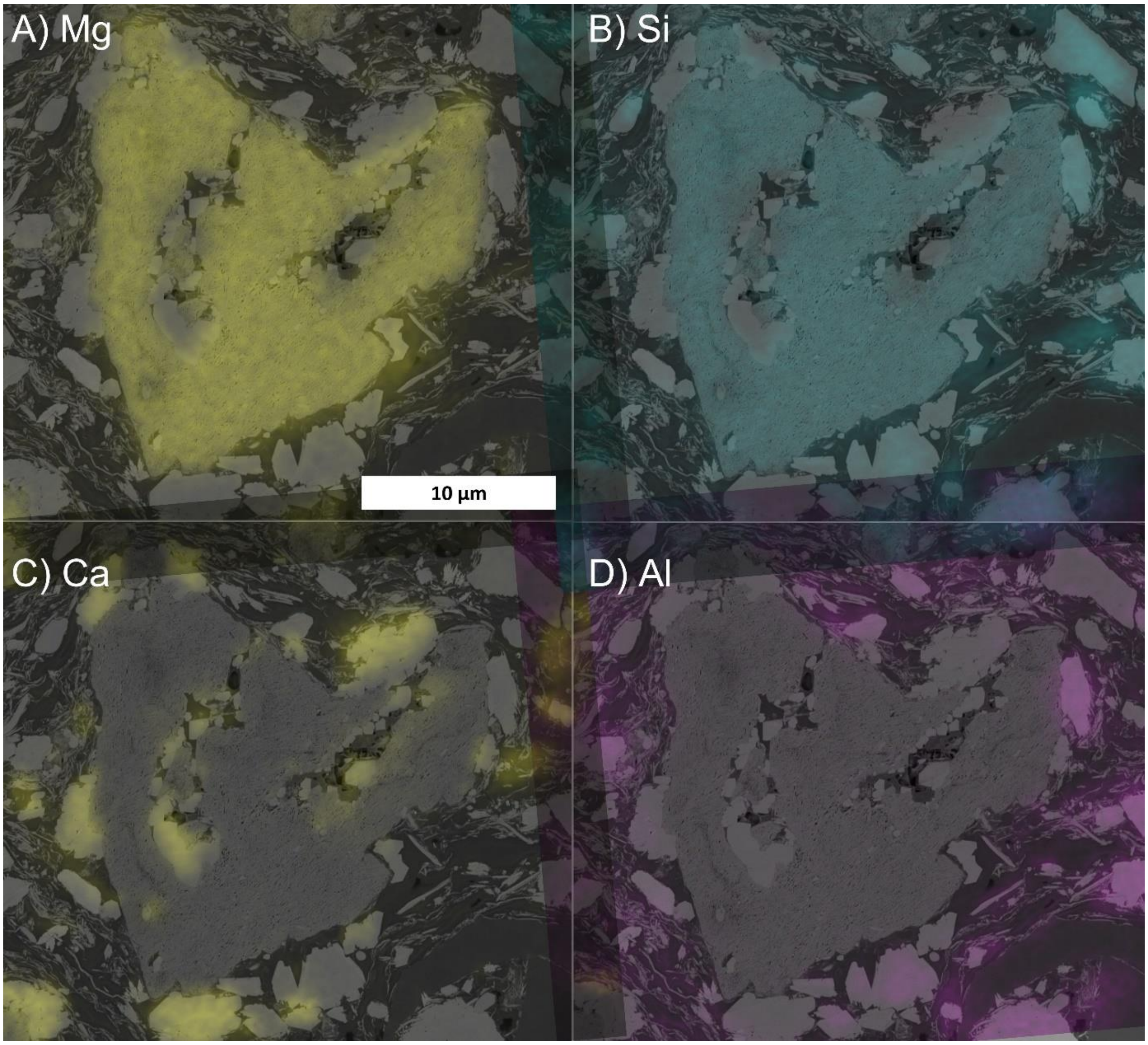

Figure S2. A fibrous mass is demonstrated to be Mg-rich in these scanning electron microscopy - energy dispersive X-ray spectroscopy (SEM-EDS) images (color) overlying SEM backscatter electron images (greyscale) of the GORD2 350×9 sample. Color intensity represents elemental concentration of the specified elements (higher intensity corresponds to higher elemental concentration). The specified elements are A) magnesium, B) silicon, C) calcium, and D) aluminum. Color difference between frames is just an arbitrary software setting and has no geological meaning.
